# Alkylative Aziridine Ring-Opening Reactions

**DOI:** 10.3390/molecules26061703

**Published:** 2021-03-18

**Authors:** Jieun Choi, Taehwan Yu, Hyun-Joon Ha

**Affiliations:** Department of Chemistry, Hankuk University of Foreign Studies, Yongin 17035, Korea; jieun3369@gmail.com (J.C.); sunjew03221@naver.com (T.Y.)

**Keywords:** aziridine, non-activated, aziridinium ion, alkylative, ring-opening, amine, regioselectivity

## Abstract

In this study, the highly strained three-membered aziridine ring was successfully activated as the aziridinium ion by alkylation of the ring nitrogen with a methyl, ethyl or allyl group, which was followed by ring opening with external nucleophiles such as acetate and azide. Such alkylative aziridine ring opening provides an easy route for the synthesis of various *N*-alkylated amine-containing molecules with concomitant introduction of an external nucleophile at either its α- or β-position.

## 1. Introduction

**Scheme 1:**
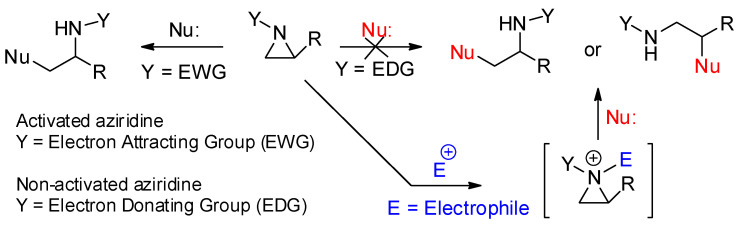
Nucleophilic ring opening of activated and non-activated aziridines.

Aziridine, a nitrogen-containing three-membered ring, has high ring strain similar to oxirane and cyclopropane [1,2,3,4,5,6,7,8,9,10,11,12]. However, the non-activated aziridine-bearing electron donating group at the ring nitrogen is relatively stable and inert toward almost all nucleophiles. It should be activated properly as an aziridinium ion or its equivalent prior to ring-opening reaction, as shown in Scheme 1 [13,14].

**Scheme 2:**
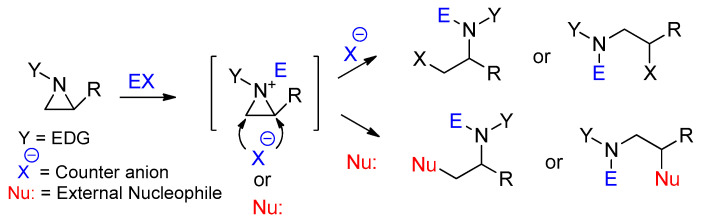
Aziridine ring opening either by a counter anion (X^−^) of an electrophile or by an external nucleophile (Nu:) added directly to the reaction vessel.

The reactivity of non-activated aziridine provides an opportunity to introduce another functional group into the ring nitrogen during activation of the aziridine ring. Electrophiles for the activation of non-activated aziridine by bond formation, including carbon–carbon and carbon–silicon, and by Lewis acid (LA) coordination with lone pair electrons at the aziridine ring nitrogen can yield aziridinium ions or their equivalents [15]. These electrophiles include haloalkanes, acyl halides, trialkylsilyl halides and most Lewis acids [16,17,18,19]. However, most aziridinium ions are quite reactive toward the counter anion (X^−^ in Scheme 2) released from reagents of electrophiles as a nucleophile to be broken down [20,21,22,23,24]. Introduction of a specific group with concomitant breakage of the ring opening by an external nucleophile is a challenging problem to be solved to boost the synthetic utility of aziridine. To make this possible, the aziridinium ion should be stable as it is a quaternary ammonium salt enduring the strain of a three-membered aziridine ring until the external nucleophile reacts for ring opening [25,26]. It means that the nucleophilicity of the counter anion from the reagent of the electrophile should be weak enough for the aziridinium ion to survive. This is a key requirement for the external nucleophile added to the reaction media that leads to ring opening at either its α- or β-position in a regioselective manner, as shown in Scheme 2.

## 2. Results and Discussion

**Scheme 3:**
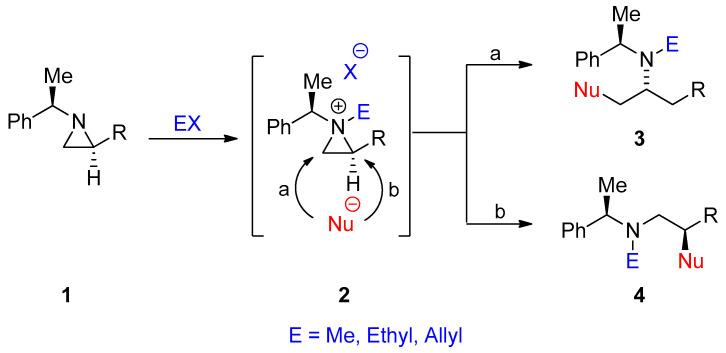
Aziridine ring opening in either ways “a” or “b”. Activation with an alkyl group from an electrophile (EX) to make the aziridinium ion in the bracket, followed by aziridine ring-opening reactions with an external nucleophile (Nu^−^) for alkylative aziridine ring opening.

Every electrophile provides an aziridinium ion with unique characteristics that leads to ring-opening reactions in distinctive regiochemical pathways; either “a” or “b” in Scheme 3, as summarized in our previous publication [27]. The ring substituent R and the nucleophile (Nu^−^) applied in the reaction vessel are also crucial to determine regiochemical pathways [27,28].

**Figure 1:**
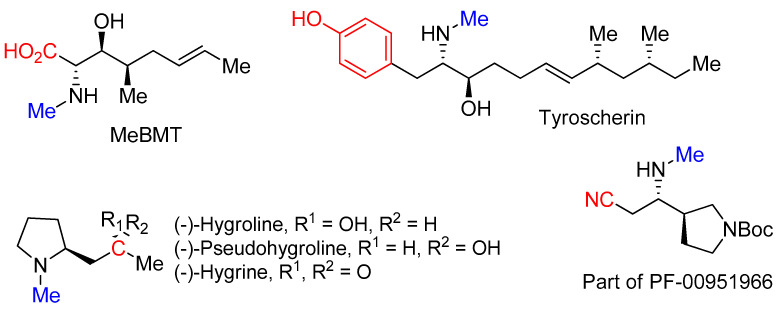
Biologically important molecules synthesized through *N*-methylative aziridine ring-opening reactions. Methyl groups (Me) shown in blue; nucleophiles shown in red.

Our previous study [27,28] showed that the reaction of aziridine (**1**) bearing a 2-methylbenzyl group as an electron-donating group (EDG) at the ring nitrogen with methyl trifluoromethylsulfonate (MeOTf) can provide stable aziridinium ions such as **2** (E = Me, X = TfO^−^) (Scheme 3). Among all electrophiles applicable to the activation of non-activated aziridine, the alkyl group is a good applicant with numerous advantages. It can provide a good chance to introduce a new alkyl group at the ring nitrogen and nucleophiles either at the α- or β-carbon of nitrogen, depending on the regiochemical pathways [29,30,31]. In a previous study, we were able to introduce a methyl group to generate the *N*-methylaziridinium ion and lead to subsequent ring openings for *N*-methylated acyclic amines in a regio- and stereoselective manner [32]. This new reaction, called the *N*-methylative aziridine ring-opening reaction, was used as a key step for the synthesis of various biologically important molecules. We succeeded in making *N*-methylative aziridine ring-opening reactions with methyl group at the nitrogen coming from MeOTf for the formation of aziridinium ions and subsequent ring opening by nucleophiles, including acetate for MeBMT [33]; protected *p*-hydroxyphenyl magnesium bromide for tyroscherin [34,35]; and nitriles for hygroline, pseudohygroline, hygrine [36] and part of PF-00951966 [37] (Figure 1). These examples showed the value of *N*-methylative aziridine ring-opening reactions.

Expansion of this reaction with alkylation other than methylation will provide a general route for the synthesis of various alkyl- or functionalized amine-containing molecules. However, there are problems associated with this new reaction that should be overcome for this reaction to be synthetically valuable. At first, the aziridinium ion derived after aziridine is alkylated should be stable enough for the external nucleophiles to be reacted. In other words, the counter anion used to form the aziridinium ion is not nucleophilic enough to break down the intermediate. Instead, an external nucleophile should be reactive enough to give a decent yield of the ring-opening reaction [37]. In addition, the regiochemical pathway should be controllable. It should be either pathway “a” or “b” to yield an α- or β-amino compound selectively. However, up to now, only *N*-methylation has been successful, while all other bigger alkyl groups have been unsuccessful, possibly due to the limitations of the small steric space to accommodate a bigger group under mild reaction conditions. This intrinsic drawback should be overcome by improving the reactivity of the incoming reactant as an electrophile-bearing non-nucleophilic leaving group. After numerous approaches, we finally succeeded in the alkylation of the ring nitrogen, including ethyl and allyl groups besides methyl, to give rise to the corresponding *N*-alkylated amines after aziridine ring opening.

At first, the ethylative aziridine ring opening was tried using the same protocol used for methylation with MeOTf as the methylating agent and with TfO^−^ as its counter anion. The ethyl group was introduced into the aziridine ring by addition of EtOTf to the substrate, followed by the external nucleophile NaOAc. As expected, the reaction medium CH_3_CN was the best to yield reaction products (**3AEa** and **4AEa**, overall yield: 49%) from 2-benzyloxymethylaziridine (**1A**), with the kinetic product as the major one and regioselectivity of 88:12 in CH_3_CN among many solvents, including DMF, THF, dioxane and CH_2_Cl_2_ (Table 1, entries 1 and 2). With an increase of the nucleophile from 1.1 to 1.5 equiv., the reaction yield was improved from 49 to 62% (entry 3). Instead of the 2-benzyloxymethyl group, 2-triethylsilyloxymethyl and 2-*t*-butyldimethylsilyloxymethyl substitution (**1B** and **1C**) afforded the expected products (**3BEa** and **4BEA**; **3CEa** and **4CEA**) in slightly better yields (64 and 72%, entries 4 and 5).

All numbered compound in Table 1 and Table 2 are classified by: (i) the second notation based on the starting substrates: **A**, R = CH_2_OBn; **B**, R = CH_2_OTES; **C**, R = CH_2_OTBS; D, R = CO_2_Et; (ii) the third notation based on the alkyl at the nitrogen N: **E** = ethyl; **A** = allyl; and (ii) the fourth notation based on the nucleophile at the carbon: **a** = OAc; **b** = N_3_.

**Scheme 4:**
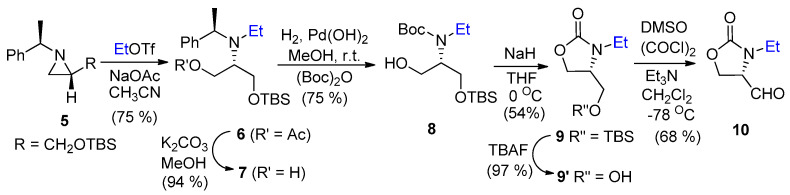
Synthesis of the key structural element of calicheamicine (**10**) bearing an ethyl group at the nitrogen from ethylative aziridine ring opening.

Such successful ethylative aziridine ring-opening reactions prompted us to realize the key structural element of calicheamicine [38] (**10**) starting from (2*S*)-2-(((tert-butyldimethylsilyl)oxy)methyl)-1-((*R*)-1-phenylethyl)aziridine (**5**) (Scheme 4). The nucleophile acetate was added to the ethylated aziridinium ion to give rise to the ring-opened product **6** in a 75% yield. This was then hydrolyzed to free alcohol (**7**). The *N*-phenylethyl group in **7** was removed by catalytic hydrogenation in the presence of (Boc)_2_O to afford **8**, followed by the treatment with a base to yield (*S*)-4-(((*tert*-butyldimethylsilyl)oxy)methyl)-3-ethyloxazolidin-2-one (**9**) [21,39,40], whose silyl group was removed by TBAF to give free alcohol (**9′**). Oxidation of alcohol (**9′**) by the Swern oxidation protocol gave the expected key structural element (**10**) for the synthesis of calicheamicine.

This result, including our previous finding, indicates that TfO^−^ is a quite unique counter anion without much nucleophilicity [25,41]. Once the ethylative aziridine ring opening has succeeded, it is of interest to add an allyl group, which has an olefin, as a potentially versatile functional group. However, allyl triflate is not stable or readily available when it is needed. The reactions with allyl triflate afforded the reaction product in poor and variable yields. Therefore, we tried to obtain the aziridinium ion with the addition of an allyl group at the ring nitrogen in different ways. Since iodine anion is a reactive nucleophile able to break the aziridine ring and its ring-opened product provides a fast equilibrium back to the aziridinium ion [8], we decided to try with allyl iodide. Thus, we observed that the iodinated product in the possible thermodynamic pathway was obtained very slowly. The reaction was not completed after more than 48 h. At that point, we decided to add AgOTf (1.05 equiv.), the silver of which would capture iodine as AgI, with the soluble triflate anion leaving the counter anion of the aziridinium ion from the allylation of aziridine. We finally found that the allylative aziridinium ion was able to be generated with ICH_2_CHCH_2_ (1.25 equiv.), AgOTf (1.05 equiv.) in CH_3_CN and external nucleophiles for ring opening (Table 2).

The reaction was performed with the starting substrate (**1A**) bearing a 2-benzyloxymethyl group as R with AgOTf, allyl iodide and sodium acetate (NaOAc) as an external nucleophile to afford the ring-opened products (**3AAa** and **4AAa**), whose combined yield was increased from 85% to 97% by using twice the amount of acetate with comparably poor regioselectivity from 50:50 to 43:57 (Table 2, entries 1 and 2). We also observed a decrease of the reaction yield from 85 to 66% by using CsOAc instead of NaOAc (entry 3). A neutral amine nucleophile is not active enough to break down the aziridinium ion to give a ring-opening product (entry 4). With NaN_3_ as the nucleophile, the reaction gave rise to the expected ring-opening products (**3AAb** and **4AAb**) without much difference in regiochemistry and reaction yield, depending on the amount of reagents added (Table 2, entries 5–7). Instead of the benzyloxymethyl group, the starting materials (**1B** and **1C**) with triethylsilyloxymethyl and *t*-butyldimethylsilyloxymethyl as substituents at the C2 of aziridine gave the expected products (**3BAa** and **4BAa** and **3CAa** and **4CAa)** with yields of 78% and 89%, respectively, by following the same procedure with NaOAc as a nucleophile (entries 8 and 9). Regioselectivity of both reactions of 99:1 was astonishing, possibly due to the steric difficulty imposed for the nucleophile to approach. We also studied reactions of starting aziridine (**1D**) bearing ethoxycarbonyl as a substituent at the C2 of aziridine with NaOAc and NaN_3_ (entries 10 and 11). Reactions with NaOAc and NaN_3_ resulted in the ring-opened products (**3DAa** and **4BAa** and **3DAb** and **4Dab**) with yields of 68% and 34%, respectively, with regioselectivities of 11:89 and 47:53, showing that the pathway b in Scheme 3 is more favorable. This regiochemical pathway b is always dominant with starting materials that have vinyl or carbonyl functional groups at the C2 of aziridine, due to the weakness of the bond between the N and C2.

**Scheme 5:**
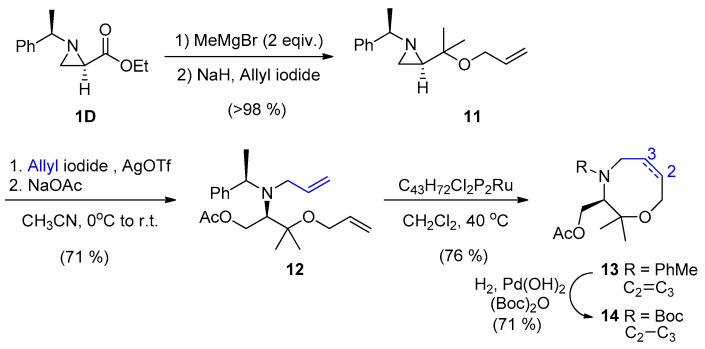
Synthesis of the unusual eight-membered ring with nitrogen and oxygen at its 1- and 4-positions from the Ring Closing Metathesis of allylated ether and allylated amine from *N*-allylative aziridine ring opening.

As a showcase of this chemistry, the allylated aziridine ring-opened product (**12**) was afforded to take advantage of the olefin at the amine nitrogen (Scheme 5). At first, the starting 2-allyloxydimethylmethylaziridine (**11**) was prepared by the addition of methyl magnesium bromide to aziridine-2-carboxylate (**1D**), followed by the allylation of the resultant free hydroxy group with allyl iodide and NaH as a base. This aziridine (**11**) was then used for an *N*-allylated aziridine ring-opening reaction with allyl iodide, AgOTf and NaOAc as a nucleophile to give the product **12** in a 71% yield. This reaction product (**12**) with two allyl olefins was cyclized in the presence of Grubbs’ catalyst to yield an eight-membered ring (**13**). By catalytic hydrogenation in the presence of (Boc)_2_O, this ring (**13**) was saturated and the phenylethyl group at the nitrogen was removed to realize a 4-azaoxo heterocycle (**14**) with valuable functional groups.

## 3. Experimental Section

### 3.1. General Information

Chiral aziridines are available from Sigma-Aldrich as reagents. They are also available from Imagene Co., Ltd. in bulk quantities. All commercially available compounds were used as received unless stated otherwise. All reactions were carried out under an atmosphere of nitrogen in oven-dried glassware with magnetic stirrer. Dichloromethane was distilled from calcium hydride. Reactions were monitored by thin layer chromatography (TLC) with 0.25 mm using a Kieselgel 60 Art 9385 from Merck (Darmstadt, Germany. Visualization was accomplished with either UV light or by immersion in solutions of ninhydrin, p-anisaldehyde or phosphomolybdic acid (PMA), followed by heating on a hot plate for about 10 s. Purification of reaction products was carried out by flash chromatography using using a Kieselgel 60 Art 9385 from Merck in Germany. (230–400 mesh). ^1^H-NMR and ^13^C-NMR spectra were obtained using a Varian Unity INOVA 400WB (400 MHz) or Bruker AVANCE III HD (400 MHz) spectrometer. Chemical shifts are reported relative to chloroform (δ = 7.26) for ^1^H NMR, chloroform (δ = 77.2) for ^13^C NMR, acetonitrile (δ = 1.94) for ^1^H NMR and acetonitrile (δ = 1.32) for ^13^C NMR (see Supplementary Materials). Data are reported as br = broad, s = singlet, d = doublet, t = triplet, q = quartet, p = quintet and m = multiplet. Coupling constants are given in Hz. Ambiguous assignments were resolved using standard one-dimensional proton decoupling experiments. Optical rotations were obtained using a Rudolph Autopol III digital polarimeter and JASCO P-2000. Optical rotation data are reported as follows: [α]^20^ (concentration c = g/100 mL, solvent). High-resolution mass spectra were recorded on a 4.7 Tesla IonSpec ESI-TOFMS, JEOL (JMS-700) and an AB Sciex 4800 Plus MALDI TOF^TM^ (a 2,5-dihydroxybenzoic acid (DHB) matrix was used to prepare samples for MS. Data were obtained in the reflector positive mode with internal standards for calibration).

### 3.2. General Procedure for the Synthesis of Ethylative Aziridine Ring-Opening Compounds

Ethyl trifluoromethanesulfonate (0.05 mL, 0.411 mmol) was added to a stirring solution of aziridine **1** (100 mg, 0.374 mmol) in dry CH_3_CN (4 mL) under N_2_ at 0 °C. After 5–10 min, NaOAc (46 mg, 0.561 mmol) was added to the solution at same temperature. After 10 min, the solution was allowed to be warmed to room temperature (RT) and stirred. After quenching by adding water (10 mL), the reaction product was extracted with CH_2_Cl_2_ (20 mL) three times. It was then dried over MgSO_4_, filtered and concentrated in vacuo. The reaction product was purified by column chromatography (1:19 = EtOAc:Hex) to provide an analytically pure product.

### 3.3. (S)-3-(benzyloxy)-2-(ethyl((R)-1-phenylethyl)amino)propyl Acetate (**3AEa**)

Yellow liquid. [α]_D_^20^ 12.1 (c = 1.0, CHCl_3_). ^1^H NMR (400 MHz, CDCl_3_) *δ* 7.45–7.10 (m, 10H), 4.33 (s, 2H), 4.24 (dd, *J* = 11.2, 6.9 Hz, 1H), 4.14 (dd, *J* = 11.2, 5.5 Hz, 1H), 4.07 (d, *J* = 6.8 Hz, 1H), 3.35–3.29 (m, 2H), 3.24 (s, 1H), 2.83–2.59 (m, 2H), 2.02 (s, 3H), 1.38 (d, *J* = 6.8 Hz, 3H), 1.01 (t, *J* = 7.0 Hz, 3H). ^13^C NMR (101 MHz, CDCl_3_) *δ* 171.10, 145.13, 138.37, 128.34, 128.08, 127.59, 127.52, 126.64, 73.07, 69.79, 64.01, 57.80, 55.60, 40.34, 21.13, 19.00, 16.05. HRMS-ESI (*m*/*z*): [M + H]^+^ calcd for C_22_H_30_NO_3_, 356.2160, found 356.2162.

### 3.4. (S)-1-(benzyloxy)-3-(ethyl((R)-1-phenylethyl)amino)propan-2-yl Acetate (**4AEa**)

Yellow liquid. ^1^H NMR (400 MHz, CDCl_3_) *δ* 7.41–7.24 (m, 10H), 5.17–5.08 (m, 1H), 4.54 (d, *J* = 5.8 Hz, 2H), 3.88 (q, *J* = 6.8 Hz, 1H), 3.65 (dd, *J* = 10.7, 3.5 Hz, 1H), 3.59 (dd, *J* = 10.7, 5.7 Hz, 1H), 2.72 (dd, *J* = 13.7, 7.0 Hz, 1H), 2.66–2.44 (m, 3H), 2.08 (s, 3H), 1.35 (d, *J* = 6.8 Hz, 3H), 1.02 (t, *J* = 7.1 Hz, 3H). ^13^C NMR (101 MHz, CDCl_3_) *δ* 170.58, 143.71, 138.02, 128.29, 127.96, 127.68, 126.57, 73.03, 71.94, 69.75, 59.04, 50.11, 44.61, 26.43, 21.27, 16.44, 12.80. HRMS-ESI (*m*/*z*): [M + H]^+^ calcd for C_22_H_30_NO_3_, 356.2160, found 356.2162.

### 3.5. (R)-2-(ethyl((R)-1-phenylethyl)amino)-3-((triethylsilyl)oxy)propyl Acetate (**3BEa**)

Yellow liquid. [α]_D_^20^ 1.1 (c = 1.0, CHCl_3_). ^1^H NMR (400 MHz, CDCl_3_) *δ* 7.38 (d, *J* = 7.3 Hz, 2H), 7.33–7.25 (m, 2H), 7.24–7.18 (m, 1H), 4.23 (dd, *J* = 11.2, 7.0 Hz, 1H), 4.16 (dd, *J* = 11.2, 5.4 Hz, 1H), 4.09 (q, *J* = 6.8 Hz, 1H), 3.43 (d, *J* = 6.3 Hz, 2H), 3.12–2.96 (m, 1H), 2.77 (dd, *J* = 13.8, 7.0 Hz, 1H), 2.68 (dd, *J* = 13.9, 7.0 Hz, 1H), 2.06 (s, 3H), 1.37 (d, *J* = 6.8 Hz, 3H), 1.02 (t, *J* = 7.0 Hz, 3H), 0.88 (dd, *J* = 9.5, 6.4 Hz, 9H), 0.48 (q, *J* = 7.9 Hz, 6H). ^13^C NMR (101 MHz, CDCl_3_) *δ* 170.98, 145.02, 138.25, 128.22, 127.96, 127.47, 126.52, 77.32, 77.00, 76.68, 72.95, 69.67, 63.89, 57.68, 55.48, 40.22, 21.02, 18.88, 15.93. HRMS-ESI (*m*/*z*): [M + H]^+^ calcd for C_21_H_38_NO_3_Si, 380.2512, found 380.2515.

### 3.6. (S)-1-(ethyl((R)-1-phenylethyl)amino)-3-((triethylsilyl)oxy)propan-2-yl Acetate (**4BEa**)

Yellow liquid. ^1^H NMR (400 MHz, CDCl_3_) δ 7.36–7.17 (m, 5H), 4.94 (dd, J = 5.9, 3.9 Hz, 1H), 3.87 (d, J = 6.8 Hz, 1H), 3.74 (dd, J = 11.1, 3.7 Hz, 1H), 3.66 (dd, J = 11.1, 5.7 Hz, 1H), 2.66 (d, J = 6.6 Hz, 1H), 2.56 (d, J = 7.0 Hz, 1H), 2.52–2.37 (m, 2H), 2.03 (s, 3H), 1.34 (d, J = 6.8 Hz, 3H), 1.25 (s, 1H), 1.00 (t, J = 7.1 Hz, 3H), 0.94 (t, J = 7.9 Hz, 9H), 0.58 (q, J = 8.0 Hz, 6H). ^13^C NMR (101 MHz, CDCl_3_) δ 173.02, 170.59, 143.71, 138.03, 128.29, 127.90, 127.61, 126.57, 77.32, 77.00, 76.68, 73.04, 71.94, 69.75, 59.12, 50.11, 44.61, 39.90, 26.37, 21.27, 16.44, 12.80. HRMS-ESI (*m*/*z*): [M + H]^+^ calcd for C_2__1_H_38_NO_3_Si, 380.2512, found 380.2515.

### 3.7. (S)-2-(((tert-butyldimethylsilyl)oxy)methyl)-1-((R)-1-phenylethyl)aziridine (**5**)

To a stirred solution of ((*S*)-1-((*R*)-1-phenylethyl)aziridin-2-yl)methanol (3.191 g, 18.016 mmol) in CH_2_Cl_2_ (40 mL) at 0 °C under an inert atmosphere of N_2_ was added t-butyldimethylsilyl chloride (4.073 mg, 24.024 mmol) and DMAP (4.402 g, 36.032 mmol). The mixture was stirred for 5 min and then warmed to RT. After stirring for 6 h at RT, the mixture was treated with saturated aqueous NaHCO_3_ solution. The organic layer was separated and the aqueous layer was extracted with CH_2_Cl_2_ (25 mL × 2). Combined organic extracts were washed with 10 mL of brine, dried over anhydrous MgSO_4_, filtered and concentrated in vacuo. The residue was purified by flash column chromatography on silica gel (EtOAc:hexanes = 1:3) to afford a pure product **5** (4.997g, 95.2%). Yellow liquid. [α]_D_^20^ +25.7 (c = 1.05, CHCl_3_). ^1^H NMR (400 MHz, CDCl_3_) δ 7.47–7.14 (m, 5H), 3.61 (dd, *J* = 10.9, 5.6 Hz, 1H), 3.44 (dd, *J* = 10.9, 5.9 Hz, 1H), 2.49 (q, *J* = 6.6 Hz, 1H), 1.79 (d, *J* = 3.4 Hz, 1H), 1.67 (dd, *J* = 6.1, 3.4 Hz, 1H), 1.45 (t, *J* = 7.1 Hz, 4H), 0.84 (s, 9H), −0.01 (d, *J* = 3.3 Hz, 6H). ^13^C NMR (101 MHz, CDCl_3_) δ 144.42, 128.17, 126.80, 126.65, 69.52, 65.71, 39.97, 32.52, 25.79, 23.08, 18.23, −5.41.

### 3.8. (S)-3-((tert-butyldimethylsilyl)oxy)-2-(ethyl((R)-1-phenylethyl)amino)propyl Acetate (**6**)

To a stirred solution of (*S*)-2-(((tert-butyldimethylsilyl)oxy)methyl)-1-((*R*)-1-phenylethyl)aziridine (**5**) (0.500 g, 1.717 mmol) in dry CH_3_CN (5.72 mL) was added ethyl trifluoromethanesulfonate (0.244 mL, 1.889 mmol) under N_2_ at 0 °C. After 5–10 min, NaOAc (0.154 g, 1.889 mmol) was added to the solution at the same temperature. After 10 min, the solution was allowed to be warmed to RT and was stirred for 12 h. Then, it was quenched by adding water (10 mL). The reaction product was extracted with CH_2_Cl_2_ (20 mL) three times. It was then dried over anhydrous MgSO_4_, filtered and concentrated in vacuo. The reaction product was purified by column chromatography (1:19 = EtOAc:Hex) to provide an analytically pure product **6** (0.411 g, 75%). Yellow liquid. [α]_D_^20^ −6.7 (c = 1.1, CHCl_3_). ^1^H NMR (400 MHz, CDCl_3_) *δ* 7.44 (d, *J* = 7.3 Hz, 2H), 7.34 (dd, *J* = 10.2, 4.8 Hz, 2H), 7.28–7.20 (m, 1H), 4.17 (d, *J* = 6.8 Hz, 1H), 4.12 (dd, *J* = 6.4, 2.1 Hz, 2H), 3.79 (dd, *J* = 10.2, 5.4 Hz, 1H), 3.72 (dd, *J* = 10.3, 6.1 Hz, 1H), 3.24–2.98 (m, 1H), 2.84–2.77 (m, 2H), 2.03 (s, 3H), 1.45 (d, *J* = 6.8 Hz, 3H), 1.07 (t, *J* = 7.1 Hz, 3H), 0.96 (s, 9H), 0.10 (d, *J* = 4.9 Hz, 6H). ^13^C NMR (101 MHz, CDCl_3_) *δ* 170.78, 145.62, 128.01, 127.55, 126.49, 63.53, 62.70, 57.69, 40.35, 25.94, 21.01, 18.65, 18.22, 16.13, −5.54. HRMS-ESI (*m*/*z*): [M + H]^+^ calcd for C_21_H_38_NO_3_Si, 380.2531, found 380.2534.

### 3.9. (S)-3-((tert-butyldimethylsilyl)oxy)-2-(ethyl((R)-1-phenylethyl)amino)propan-1-ol (**7**)

To a stirred solution of *(S)*-3-((tert-butyldimethylsilyl)oxy)-2-(ethyl(*(R)*-1-phenylethyl)amino)propyl acetate (**6**) (1.30 g, 3.428 mmol) in MeOH (10 mL) at 0 °C under an inert atmosphere of N_2_ was added potassium carbonate (0.710 g, 5.142 mmol). The mixture was stirred for 10 min. The mixture was further stirred for 3 h at RT and was treated with saturated aqueous NaHCO_3_ solution. The organic layer was separated and the aqueous layer was extracted with CH_2_Cl_2_ (25 mL × 2). Combined organic extracts were washed with 10 mL of brine, dried over anhydrous MgSO_4_, filtered and concentrated in vacuo. The residue was purified by flash column chromatography on silica gel (EtOAc:hexanes = 1:3) to afford a pure product **7** (0.841 g, 94%). Yellow liquid. [α]_D_^20^ −53.0 (c = 1.05, CHCl_3_). ^1^H NMR (400 MHz, CDCl_3_) *δ* 7.43–7.18 (m, 5H), 4.14 (q, *J* = 6.8 Hz, 1H), 3.78 (dd, *J* = 10.3, 5.3 Hz, 1H), 3.64–3.46 (m, 2H), 3.33 (t, *J* = 10.0 Hz, 1H), 3.15–3.02 (m, 1H), 2.90 (s-br, 1H), 2.70 (q, *J* = 7.2 Hz, 2H), 1.42 (d, *J* = 6.8 Hz, 3H), 1.00 (t, *J* = 7.1 Hz, 3H), 0.89 (s, 9H), 0.05 (s, 6H). ^13^C NMR (101 MHz, CDCl_3_) *δ* 144.94, 128.29, 127.37, 126.81, 62.13, 60.14, 59.32, 56.51, 39.51, 25.75, 18.25, 17.36, 15.45, −5.59. HRMS-ESI (*m*/*z*): [M + H]^+^ calcd for C_19_H_36_NO_2_Si, 338.2402, found 338.2405.

### 3.10. (S)-tert-butyl (1-((tert-butyldimethylsilyl)oxy)-3-hydroxypropan-2-yl)(ethyl)-carbamate (**8**)

To a stirred solution of *(S)*-3-((*tert*-butyldimethylsilyl)oxy)-2-(ethyl(*(R)*-1-phenylethyl)amino)propyl acetate (**7**) (1.130 g 2.9980 mmol) in MeOH (12 mL) was added Pd(OH)_2_ (0.052 g, 50% wet) and di-tert-butyl dicarbonate (0.975 g, 4.469 mmol) at RT under H_2_. After the reaction was completed, the reaction mixture was filtered with methanol and then concentrated in vacuo. The residue was purified by flash column chromatography on silica gel to afford the pure product **8** (0.612 g, 75%). Yellow liquid. [α]_D_^20^ 3.1 (c = 1.0, CHCl_3_). ^1^H NMR (400 MHz, CDCl_3_) *δ* 3.96 (d, *J* = 7.3 Hz, 1H), 3.89–3.79 (m, 3H), 3.75 (s, 1H), 3.60 (s, 1H), 3.36–3.29 (m, 1H), 3.25–3.05 (m, 1H), 1.46 (s, 9H), 1.12 (t, *J* = 6.8 Hz, 3H), 0.89 (s, 9H), 0.06 (s, 6H). ^13^C NMR (101 MHz, CDCl_3_) *δ* 171.06, 79.64, 63.17, 61.83, 60.31, 43.08, 28.33, 25.72, 20.93, 18.09, 14.06, −5.60. HRMS-ESI (*m*/*z*): [M + H]^+^ calcd for C_16_H_36_NO_4_Si, 334.2310, found 380.2313.

### 3.11. (S)-4-(((tert-butyldimethylsilyl)oxy)methyl)-3-ethyloxazolidin-2-one (**9**)

To a stirred solution of *(S)*-3-((tert-butyldimethylsilyl)oxy)-2-(ethylamino)propan-1-ol (**8**) (0.424 g, 1.818 mmol) in dry THF (9.09 mL) was added NaH (52 mg, 0.455 mmol) under N_2_ at 0 °C, and after 1 h, was quenched by adding water (10 mL). The reaction product was extracted with CH_2_Cl_2_ (50 mL) three times. Then, it was dried over anhydrous MgSO_4_, filtered and concentrated in vacuo. The reaction product was purified by column chromatography to provide an analytically pure product **9** (0.185 g, 54%). Yellow liquid. [α]_D_^20^ −1.7 (c = 1.05, CHCl_3_). ^1^H NMR (400 MHz, CDCl_3_) *δ* 4.23 (ddd, *J* = 13.8, 6.4, 4.6 Hz, 1H), 4.00 (ddd, *J* = 10.7, 6.9, 5.2 Hz, 1H), 3.81 (dd, *J* = 8.5, 4.2 Hz, 1H), 3.69–3.53 (m, 2H), 3.52–3.35 (m, 1H), 3.08 (ddd, *J* = 6.6, 5.9, 3.2 Hz, 1H), 1.13–1.00 (m, 3H), 0.79 (s, 9H), -0.01 (s, 6H). ^13^C NMR (101 MHz, CDCl_3_) *δ* 157.99, 77.89, 77.16, 76.69, 64.29, 62.20, 55.60, 36.87, 25.43, 17.79, 12.62, -5.83. HRMS-ESI (*m*/*z*): [M + H]^+^ calcd for C_12_H_26_NO_3_Si, 260.1605, found 260.1607.

### 3.12. (R)-3-ethyl-4-(hydroxymethyl)oxazolidin-2-one (**9′**)

To a stirred solution of *(S)*-4-(((tert-butyldimethylsilyl)oxy)methyl)-3-ethyloxazolidin-2-one (**9**) (0.543 g, 2.095 mmol) in dry THF (6.98 mL) at 0 °C was added TBAF (2.304 mL 1.0 M in THF, 2.305 mmol) and reaction mixture was stirred for 3 h. Reaction mixture was quenched with saturated NH_4_Cl solution and then it was extracted with EtOAc (3 × 10 mL). Combined organic layer was dried over anhydrous Mg_2_SO_4_. The reaction product was purified by column chromatography (1:9 = methanol:ethyl acetate) to provide an analytically pure product **9′** (0.294 g, 97%). Yellow liquid. [α]_D_^20^ 40.5 (c = 1.0, CHCl_3_). ^1^H NMR (400 MHz, CDCl_3_) *δ* 4.39 (t, *J* = 8.9 Hz, 1H), 4.26 (dd, *J* = 8.7, 5.9 Hz, 1H), 4.02–3.89 (m, 1H), 3.86–3.81 (m, 1H), 3.76–3.64 (m, 1H), 3.62–3.53 (m, 1H), 3.25–3.17 (m, 1H), 1.21 (t, *J* = 7.2 Hz, 3H). ^13^C NMR (101 MHz, CDCl_3_) *δ* 158.68, 64.65, 60.48, 55.69, 36.70, 12.42. HRMS-ESI (*m*/*z*): [M + H]^+^ calcd for C_6_H_12_NO_3_, 146.0724, found 146.0726.

### 3.13. (S)-3-ethyl-2-oxooxazolidine-4-carbaldehyde (**10**)

To a stirred dimethyl sulfoxide (0.244 mL, 3.447 mmol) in dry CH_2_Cl_2_ (4.60 mL) was added oxalyl chloride (0.141 mL, 1.654 mmol) under N_2_ at −78 °C. The reaction mixture was stirred for 1 h. Then, *(R)*-3-ethyl-4-(hydroxymethyl)oxazolidin-2-one (**9′**) (0.200 g, 1.379 mmol) was added to the solution under the same condition. After 1 h, triethylamine (0.576 mL, 4.136 mmol) was added and stirred for 40 min. After quenching the reaction, the reaction product was extracted with CH_2_Cl_2_ (3 × 10 mL). Combined organic layer was dried over Na_2_SO_4_. The reaction product was purified by column chromatography (2:1 = ethyl acetate:hexane) to provide an analytically pure product **10** (0.134 g, 68%). Yellow liquid. ^1^H NMR (400 MHz, CDCl_3_) δ 9.68 (d, *J* = 0.7 Hz, 1H), 3.94–3.90 (m, 1H), 3.84–3.79 (m, 1H), 3.74–3.65 (m, 1H), 3.60–3.51 (m, 1H), 3.24–3.15 (m, 1H), 1.19 (t, *J* = 7.2 Hz, 3H). ^13^C NMR (101 MHz, CDCl_3_) *δ* 197.22, 158.67, 64.65, 55.68, 36.70, 12.42. HRMS-ESI (*m*/*z*): [M + H]^+^ calcd for C_6_H_10_NO_3_, 144.0612, found 144.0615.

### 3.14. General Procedure for the Synthesis of Allylative Aziridine Ring Opening Compounds

Silver trifluoromethanesulfonate (0.07 g, 0.393 mmol) and allyl iodide (0.05 mL, 0.468 mmol) were added to a stirring solution of aziridine **1** (100 mg, 0.374 mmol) in dry CH_3_CN (4 mL) under N_2_ at 0 °C. After 5–10 min, nucleophile (1.1 equiv.) was added to the solution at same condition. After 10 min, the solution was allowed to be warmed to RT and was stirred. After quenching by adding water (10 mL), the reaction product was extracted with CH_2_Cl_2_ (20 mL) three times. It was then dried over anhydrous MgSO_4_, filtered and concentrated in vacuo. The reaction product was purified by column chromatography (1:19 = ethyl acetate:hexane) to provide an analytically pure product.

### 3.15. (S)-2-(allyl((R)-1-phenylethyl)amino)-3-(benzyloxy)propyl Acetate (**3AAa**)

Yellow liquid. [α]_D_^20^ 19.4 (c = 1.0, CHCl_3_) ^1^H NMR (400 MHz, CDCl_3_) *δ* 7.42–7.07 (m, 10H), 5.97–5.69 (m, 1H), 5.17 (dd, *J* = 17.2, 1.7 Hz, 1H), 5.04 (dd, *J* = 10.1, 1.6 Hz, 1H), 4.30 (s, 2H), 4.28–4.19 (m, 1H), 4.19–4.02 (m, 2H), 3.36 (dd, *J* = 15.2, 6.0 Hz, 1H), 3.31–3.20 (m, 4H), 2.00 (s, 3H), 1.36 (d, *J* = 6.9 Hz, 3H). ^13^C NMR (101 MHz, CDCl_3_) *δ* 170.74, 144.23, 138.72, 138.09, 128.02, 127.91, 127.38, 126.53, 115.64, 72.84, 69.29, 63.58, 57.53, 54.99, 49.51, 20.90, 18.59. HRMS-ESI (*m*/*z*): [M + H]^+^ calcd for C_23_H_30_NO_3_, 368.2102, found 368.2106.

### 3.16. (S)-2-(allyl((R)-1-phenylethyl)amino)-3-((tert-butyldimethylsilyl)oxy)propyl Acetate (**3CAa**)

Yellow liquid. [α]_D_^20^ 10.8 (c = 1.0, CHCl_3_). ^1^H NMR (400 MHz, CDCl_3_) *δ* 7.37 (d, *J* = 7.2 Hz, 2H), 7.33–7.27 (m, 2H), 7.23-7.19 (m, 1H), 5.86-5.76 (m, 1H), 5.24–5.10 (m, 1H), 5.05 (dd, *J* = 10.1, 1.7 Hz, 1H), 4.13 (q, *J* = 6.8 Hz, 1H), 4.02 (d, *J* = 6.4 Hz, 2H), 3.72 (dd, *J* = 10.3, 5.5 Hz, 1H), 3.66 (dd, *J* = 10.3, 5.9 Hz, 1H), 3.34 (d, *J* = 6.1 Hz, 2H), 3.10 (t, *J* = 5.9 Hz, 1H), 1.98 (s, 3H), 1.38 (d, *J* = 6.8 Hz, 3H), 0.88 (s, 9H), 0.03 (d, *J* = 4.4 Hz, 6H). ^13^C NMR (101 MHz, CDCl_3_) *δ* 170.96, 145.05, 139.10, 128.05, 127.57, 126.56, 115.71, 63.44, 62.46, 57.30, 56.88, 49.84, 25.85, 21.09, 18.23, −5.54. HRMS-ESI (*m*/*z*): [M + H]^+^ calcd for C_22_H_38_NO_3_Si, 392.2563, found 392.2565.

### 3.17. (R)-ethyl 3-acetoxy-2-(allyl((R)-1-phenylethyl)amino)propanoate (**3DAa**)

Yellow liquid. [α]_D_^20^ 18.9 (c = 1.0, CHCl_3_). ^1^H NMR (400 MHz, CDCl_3_) *δ* 7.39–7.26 (m, 4H), 7.25–7.18 (m, 1H), 5.78 (dd, *J* = 17.2, 10.2 Hz, 1H), 5.15 (dd, *J* = 17.2, 1.8 Hz, 1H), 5.12–5.05 (m, 2H), 4.21–4.12 (m, 2H), 4.00 (q, *J* = 6.8 Hz, 1H), 3.12 (d, *J* = 6.4 Hz, 2H), 2.99 (dd, *J* = 14.1, 4.5 Hz, 1H), 2.89 (dd, *J* = 14.1, 7.2 Hz, 1H), 2.10 (s, 3H), 1.35 (d, *J* = 6.8 Hz, 3H), 1.25 (t, *J* = 7.1 Hz, 3H). ^13^C NMR (101 MHz, CDCl_3_) *δ* 170.15, 169.28, 143.05, 136.27, 127.88, 127.56, 126.65, 116.98, 72.30, 61.05, 58.54, 53.69, 50.03, 20.60, 15.17, 13.95. HRMS-ESI (*m*/*z*): [M + H]^+^ calcd for C_18_H_26_NO_4_, 320.1812, found 320.1815.

### 3.18. (R)-ethyl 2-(allyl((R)-1-phenylethyl)amino)-3-azidopropanoate (**3DAb**)

Yellow liquid. [α]_D_^20^ 24.8 (c = 1.0, CHCl_3_). ^1^H NMR (400 MHz, CDCl_3_) *δ* 7.35–7.28 (m, 4H), 7.27–7.21 (m, 1H), 5.79–5.71 (m, 1H), 5.19–5.12 (m, 1H), 5.10 (dd, *J* = 10.1, 1.5 Hz, 1H), 4.38–4.20 (m, 2H), 4.18 (dd, *J* = 10.1, 5.4 Hz, 1H), 3.95 (q, *J* = 6.9 Hz, 1H), 3.27 (dd, *J* = 13.6, 10.1 Hz, 1H), 3.05 (t, *J* = 6.0 Hz, 2H), 2.78 (dd, *J* = 13.6, 5.4 Hz, 1H), 1.37 (d, *J* = 6.9 Hz, 3H), 1.32 (t, *J* = 7.1 Hz, 3H). ^13^C NMR (101 MHz, CDCl_3_) *δ* 169.67, 142.48, 136.55, 128.17, 127.71, 127.02, 117.33, 61.83, 59.90, 55.18, 54.68, 54.59, 54.41, 16.01, 14.14. HRMS-ESI (*m*/*z*): [M + H]^+^ calcd for C_16_H_23_N_4_O_2_, 303.1714, found 303.1716.

### 3.19. (R)-2-(allyl((R)-1-phenylethyl)amino)-3-(allyloxy)-3-methylbutyl acetate (**12**)

To a stirred solution of *(R)-*2-(2-(allyloxy)propan-2-yl)-1-(*(R)*-1-phenylethyl)aziridine (**11**) (0.858 g, 3.951 mmol) in dry CH_3_CN (13.17 mL) was added silver trifluoromethanesulfonate (1.116 g, 4.346 mmol) and allyl iodide (0.405 mL, 4.346 mmol) under N_2_ at 0 °C. After 5–10 min, NaOAc (0.356 g, 4.346 mmol) was added to the solution. After 10 min, the solution was allowed to be warmed to RT and was stirred for 3 h. After quenching by adding water (10 mL), the reaction product was extracted with CH_2_Cl_2_ (20 mL) three times. It was then dried over anhydrous MgSO4, filtered and concentrated in vacuo. The reaction product was purified by column chromatography (1:9 = EtOAc:Hex) to provide an analytically pure product **12** (0.525 g, 71%). Yellow liquid. [α]_D_^20^ 17.6 (c = 1.0, CHCl_3_). ^1^H NMR (400 MHz, CDCl_3_) *δ* ^1^H NMR (400 MHz, CDCl_3_) δ 7.36–7.24 (m, 4H), 7.23–7.16 (m, 1H), 5.97–5.75 (m, 2H), 5.17 (dq, *J* = 17.2, 1.8 Hz, 1H), 5.12–5.00 (m, 3H), 4.57 (dd, *J* = 11.9, 4.3 Hz, 1H), 4.37 (dd, *J* = 11.9, 7.1 Hz, 1H), 4.29 (q, *J* = 6.9 Hz, 1H), 3.79 (dt, *J* = 5.0, 1.6 Hz, 2H), 3.52 (ddt, *J* = 14.6, 4.7, 1.6 Hz, 1H), 3.40 (dd, *J* = 14.6, 8.1 Hz, 1H), 2.96 (dd, *J* = 7.1, 4.3 Hz, 1H), 2.08 (s, 3H), 1.36 (d, *J* = 6.9 Hz, 3H), 1.02 (s, 3H), 0.98 (s, 3H). ^13^C NMR (101 MHz, CDCl_3_) *δ* 170.96, 144.49, 138.91, 134.71, 128.05, 127.53, 126.64, 116.60, 115.71, 71.90, 69.40, 63.84, 57.73, 55.19, 49.61, 21.07, 18.73. HRMS-ESI (*m*/*z*): [M + H]^+^ calcd for C_21_H_32_NO_3_, 346.2373, found 346.2375.

### 3.20. ((R,Z)-2,2-dimethyl-4-((R)-1-phenylethyl)-3,4,5,8-tetrahydro-2H-1,4-oxazocin-3-yl)methyl Acetate (**13**)

To a solution of *(R)*-2-(allyl(*(R)*-1-phenylethyl)amino)-3-(allyloxy)-3-methylbutyl acetate (**12**) (0.150 g, 0.473 mmol) in CH_2_Cl_2_ (120 mL) was added benzylidene-bis(tricyclohexylphosphine)dichlororuthenium (0.039 g, 0.048 mmol). The reaction mixture was then stirred at reflux for 8 h. After completion of the reaction (monitored by TLC), solvent was evaporated under reduced pressure. Purification of the crude residue by silica gel column chromatography (ethyl acetate:hexane = 1:9) afforded **13** (71 mg, 76%). Yellow liquid. [α]_D_^20^ -3.5 (c = 1.0, CHCl_3_). ^1^H NMR (400 MHz, CDCl_3_) *δ* 7.35–7.28 (m, 4H), 7.24–7.20 (m, 1H), 5.60–5.47 (m, 1H), 5.45–5.33 (m, 1H), 4.51–4.45 (m, 2H), 4.26 (dd, *J* = 12.2, 6.9 Hz, 1H), 4.14–4.05 (m, 1H), 4.01 (q, *J* = 6.8 Hz, 1H), 3.66 (dd, *J* = 16.2, 6.3 Hz, 1H), 3.10–3.04 (m, 2H), 1.86 (s, 3H), 1.46 (s, 3H), 1.38 (d, *J* = 6.8 Hz, 3H), 1.24 (s, 3H). ^13^C NMR (101 MHz, CDCl_3_) *δ* 170.84, 145.02, 129.91, 129.50, 128.00, 127.73, 126.66, 77.99, 63.73, 62.89, 62.56, 61.73, 43.80, 27.60, 24.37, 20.88, 20.48. HRMS-ESI (*m*/*z*): [M + H]^+^ calcd for C_19_H_28_NO_3_, 318.2014, found 318.2017.

### 3.21. (R)-tert-butyl 3-(acetoxymethyl)-2,2-dimethyl-1,4-oxazocane-4-carboxylate (**14**)

To a stirred solution of ((*R*,*Z*)-2,2-dimethyl-4-((*R*)-1-phenylethyl)-3,4,5,8-tetrahydro-2H-1,4-oxazocin -3-yl)methyl acetate (**13**) (306 mg, 0.965 mmol) in MeOH (9.65 mL) was added Pd(OH)_2_ (634 mg, 1.158 mmol, 50% wet) and the resultant reaction mixture was stirred at RT under atmospheric power of H_2_ gas. After 30 min, (Boc)_2_O (421 mg, 1.929 mmol) was added to the reaction flask at RT. The reaction mixture was stirred for 1.0 h, filtered with MeOH, and concentrated in vacuo and purified by silica gel chromatography to get the desired product **14** (215 mg, 71%). Yellow liquid. ^1^H NMR (400 MHz, CDCl_3_) *δ* 4.82 (d, *J* = 9.7 Hz, 1H), 4.34 (dd, *J* = 11.2, 4.0 Hz, 1H), 4.09 (t, *J* = 10.1 Hz, 1H), 3.85–3.79 (m, 1H), 3.33–3.29 (m, 2H), 2.05 (d, *J* = 3.6 Hz, 3H), 1.64 (s, 1H), 1.45 (s, 9H), 1.36–1.30 (m, 3H), 1.22 (s, 3H), 1.18 (s, 3H). ^13^C NMR (101 MHz, CDCl_3_) *δ* 170.84, 152.05, 79.93, 77.99, 70.12, 63.18, 53.74, 43.80, 28.59, 27.60, 25.91, 24.37, 20.88, 20.42. HRMS-ESI (*m*/*z*): [M + H]^+^ calcd for C_16_H_30_NO_5_, 316.2034, found 316.2037.

## 4. Conclusions

In conclusion, the highly strained three-membered aziridine ring is successfully activated as the aziridinium ion through alkylation of the ring nitrogen, including with methyl, ethyl and allyl groups, followed by ring opening with nucleophiles such as acetate and azide. This so-called *N*-alkylative aziridine ring opening may provide an easy route for the synthesis of various *N*-alkylated amine-containing molecules with concomitant introduction of an external nucleophile at either its α- or β-position.

## Figures and Tables

**Table 1:**
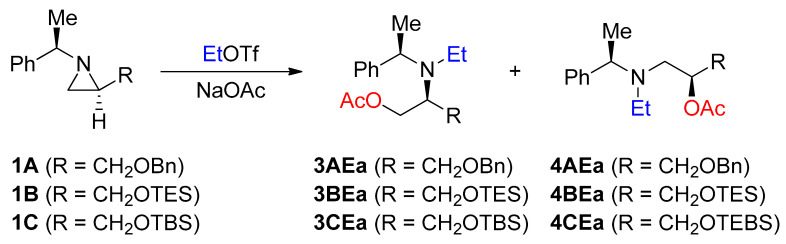
Ethylative aziridine ring-opening reactions with EtOTf in CH_3_CN ^a^.

Entry	R	MNu (equiv.)	Solvent	Rxn (h)	Yield (%) ^b^	3:4
1	CH_2_OBn	NaOAc (1.1)	CH_3_CN	6	49%	88:12
2	CH_2_OBn	NaOAc (1.1)	DMF	12	12%	62:38
3	CH_2_OBn	NaOAc (1.5)	CH_3_CN	6	62%	96:4
4	CH_2_OTES	NaOAc (1.5)	CH_3_CN	12	64%	96:4
5	CH_2_OTBS	NaOAc (1.5)	CH_3_CN	12	72%	84:16

^a^ Reaction conditions: aziridine **1** (0.374 mmol), ethyl trifluoromethanesulfonate (0.411 mmol), NaOAc (0.561 mmol) and solvent (4 mL), under a nitrogen atmosphere. ^b^ Isolated yield.

**Table 2:**
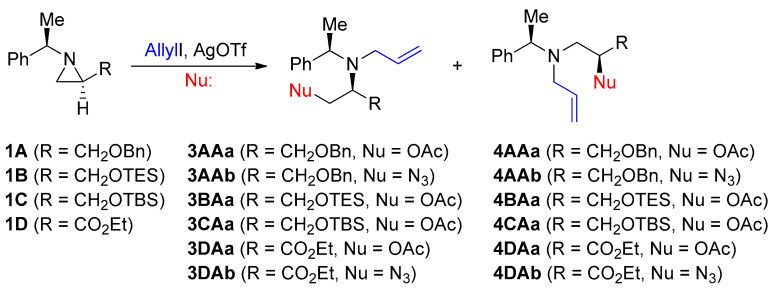
Allylative aziridine ring-opening reactions with ICH_2_CHCH_2_ (1.25 equiv.) and AgOTf (1.05 equiv.) in CH_3_CN ^a^.

Entry	R	MNu (Equiv.)	Rxn (h)	Yield (%) ^b^	3:4
1	CH_2_OBn	NaOAc (1.5)	8	85%	50:50
2	CH_2_OBn	NaOAc (3.0)	8	97%	43:57
3	CH_2_OBn	CsOAc (1.5)	24	66%	60:40
4	CH_2_OBn	BnNH_2_ (1.1)	24	-	-
5	CH_2_OBn	NaN_3_ (1.1)	12	59	50:50
6	CH_2_OBn	NaN_3_ (2.0)	8	51	50:50
7	CH_2_OBn	NaN_3_ (3.0)	8	48	50:50
8	CH_2_OTES	NaOAc (1.1)	8	78	99:1
9	CH_2_OTBS	NaOAc (1.1)	8	89	99:1
10	C(=O)OEt	NaOAc (1.1)	12	68	11:89
11	C(=O)OEt	NaN_3_ (1.1)	24	34	47:53

^a^ Reaction conditions: aziridine **1** (0.374 mmol), allyl iodide (0.468 mmol), AgOTf (0.393 mmol) and solvent (4 mL), under a nitrogen atmosphere. ^b^ Isolated yield.

## Data Availability

The data presented in this study are available on request from the corresponding author.

## Supplementary Materials

All analytical data of compounds other than the representative example are reported along the copies of ^1^H and ^13^C NMR spectra.

## Sample Availability

Samples of the compounds in this paper are available on your request from the authors.
