# Supplementary material for: Alkylative Aziridine Ring-Opening Reactions
Source: Molecules. 2021 Mar 18;26(6):1703. doi: 10.3390/molecules26061703 (PMC8003214; doi:10.3390/molecules26061703)

Supporting Information

Alkylative aziridine ring-opening reactions

Jieun Choi, Taehwan Yu and Hyun-Joon Ha*

Department of Chemistry, Hankuk University of Foreign Studies, Yongin 17035, South Korea,

E-mail: hjha@hufs.ac.kr (H.-J. Ha)

Table of Contents:

Copies of ^{1}H and ^{13}C NMR spectra……………………………………….............. p.S03-S38

^{1}H and ^{13}C NMR Spectra

^{1}H NMR spectrum of (S)-3-(benzyloxy)-2-(ethyl((R)-1-phenylethyl)amino)propyl acetate (3AEa):

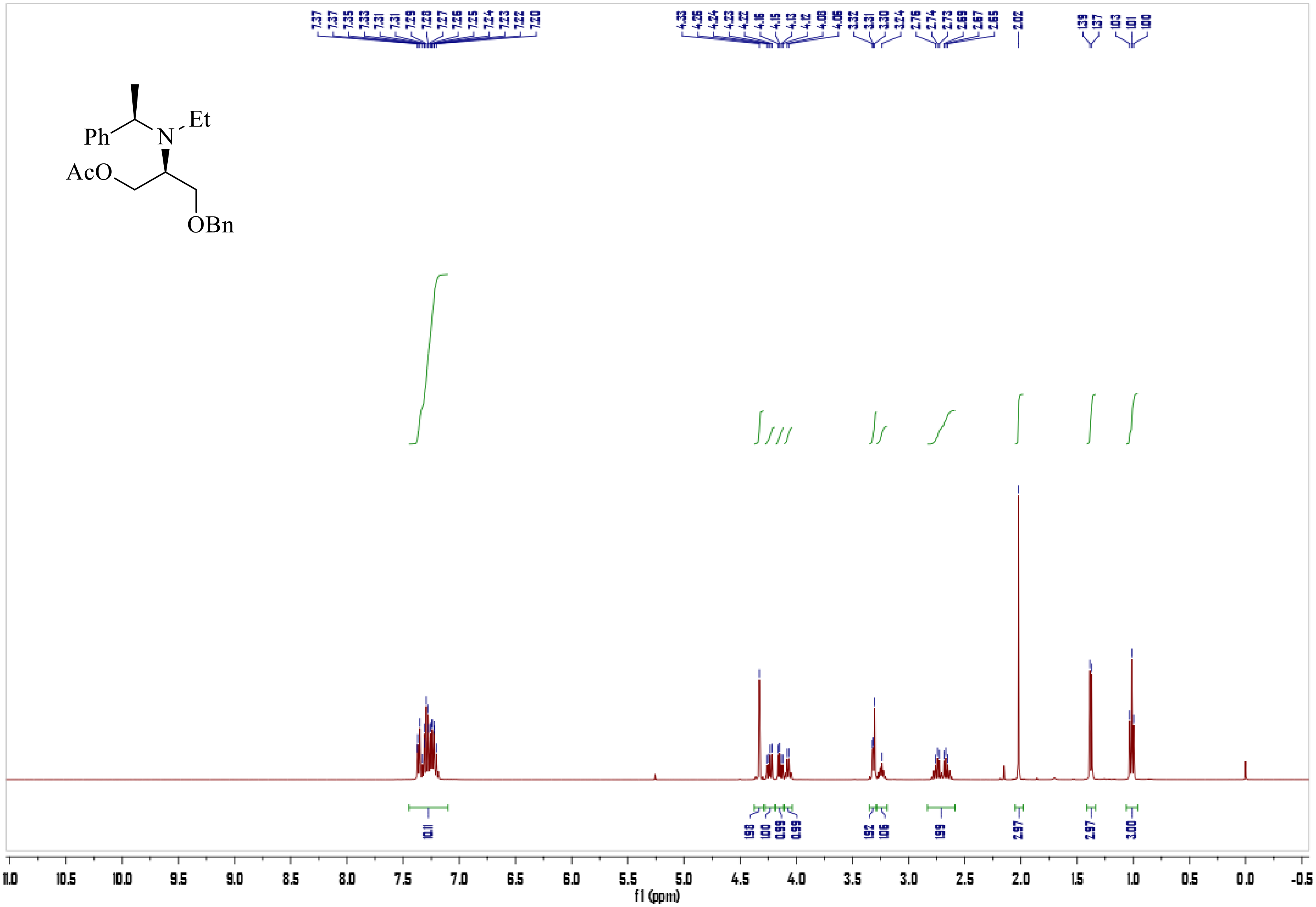

^{13}C NMR spectrum of (S)-3-(benzyloxy)-2-(ethyl((R)-1-phenylethyl)amino)propyl acetate (3AEa):

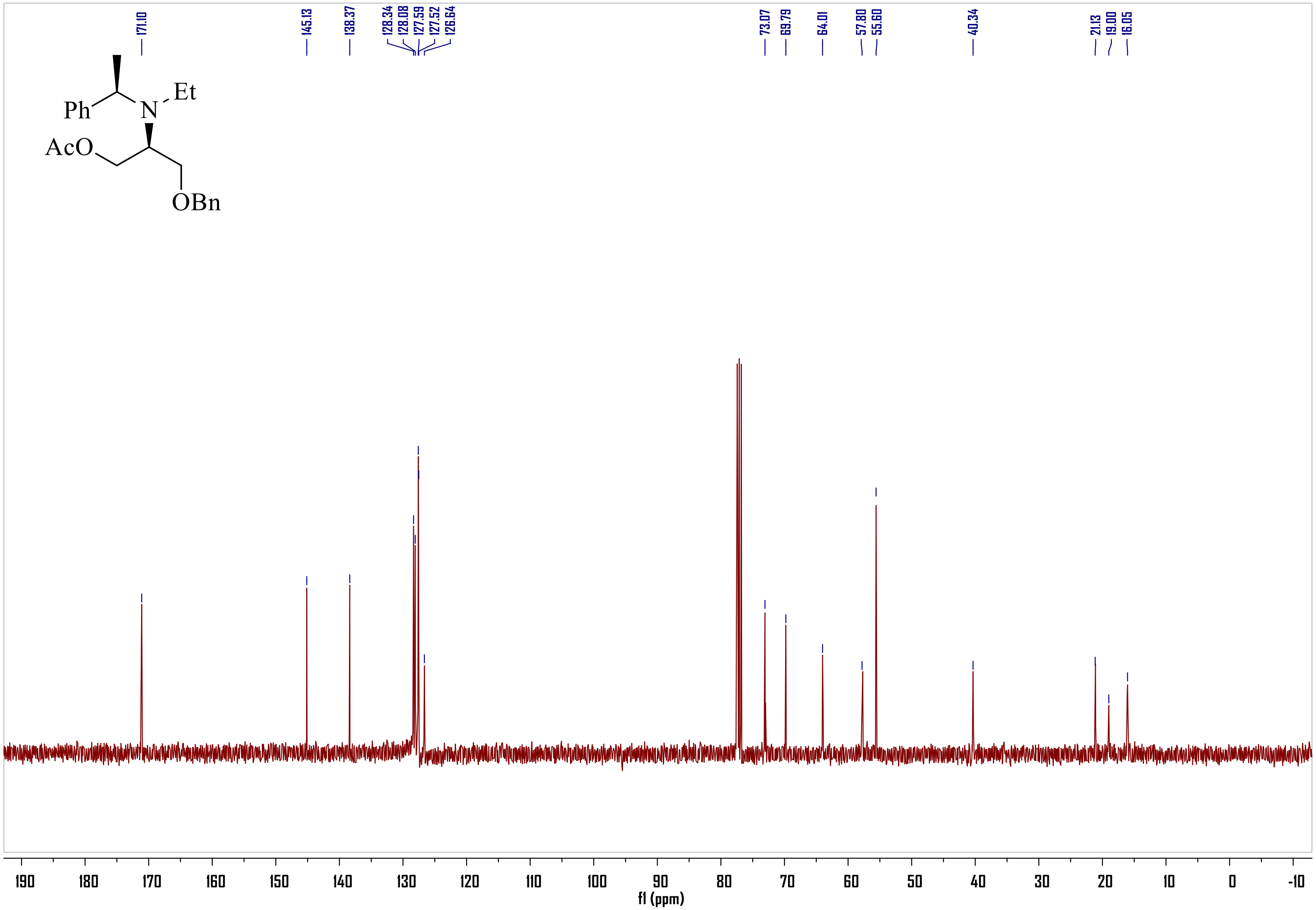

1H NMR spectrum of (S)-1-(benzyloxy)-3-(ethyl((R)-1-phenylethyl)amino)propan-2-yl acetate (4AEa):

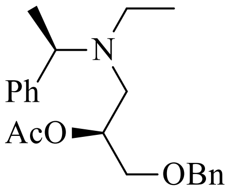

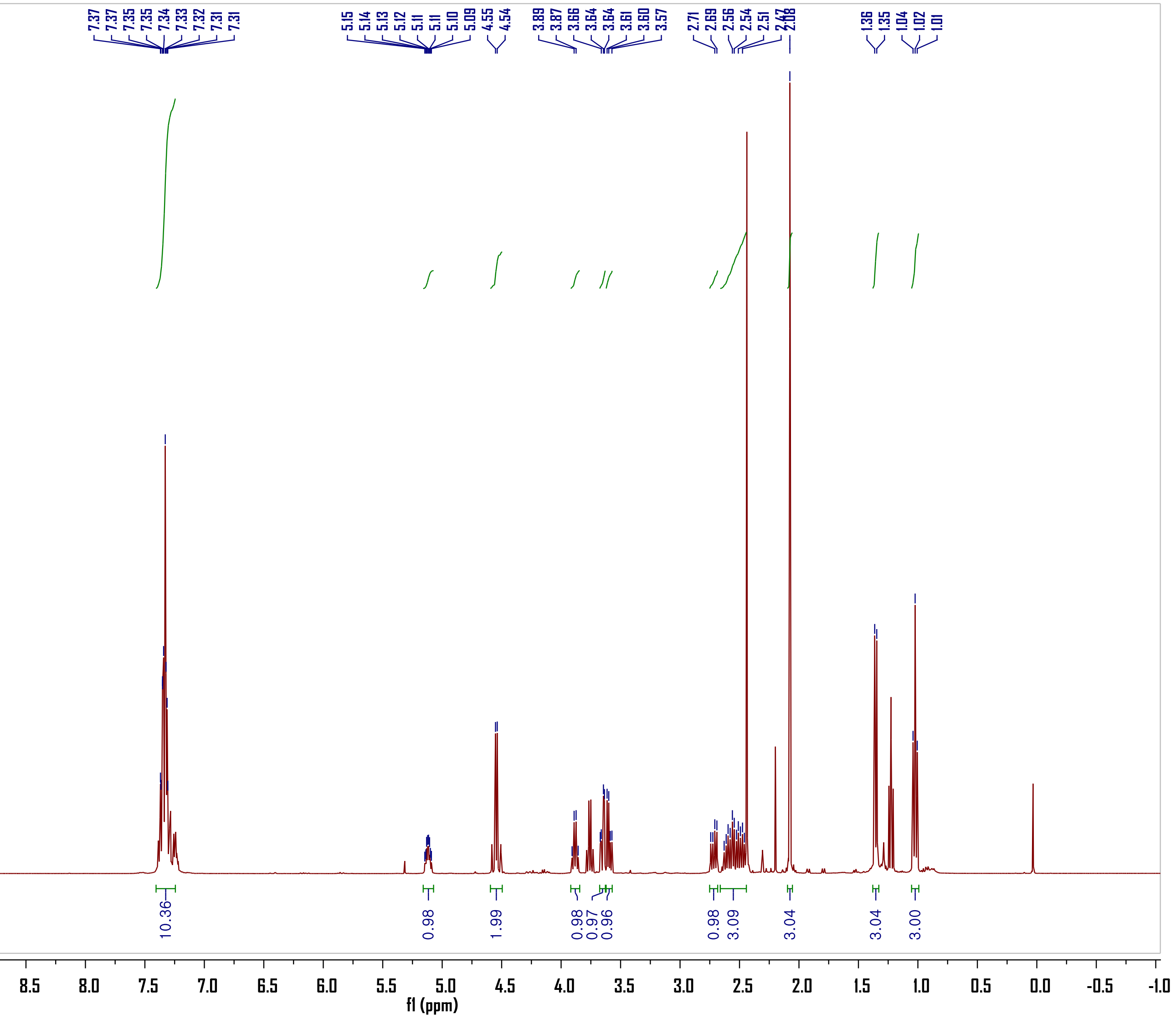

^{13}C NMR spectrum of (S)-1-(benzyloxy)-3-(ethyl((R)-1-phenylethyl)amino)propan-2-yl acetate (4AEa):

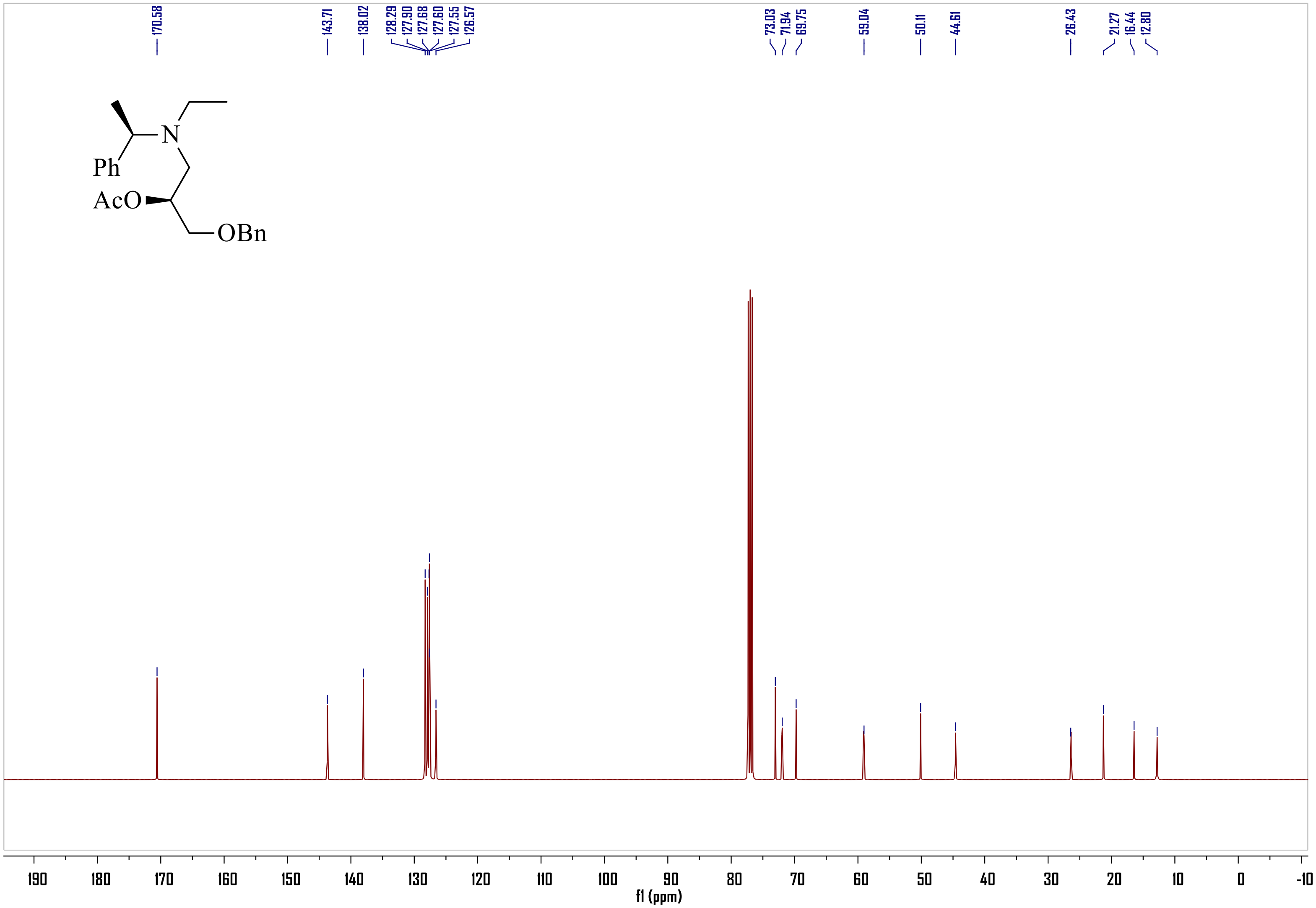

^{1}H NMR spectrum of (R)-2-(ethyl((R)-1-phenylethyl)amino)-3-((triethylsilyl)oxy)propyl acetate (3BEa):

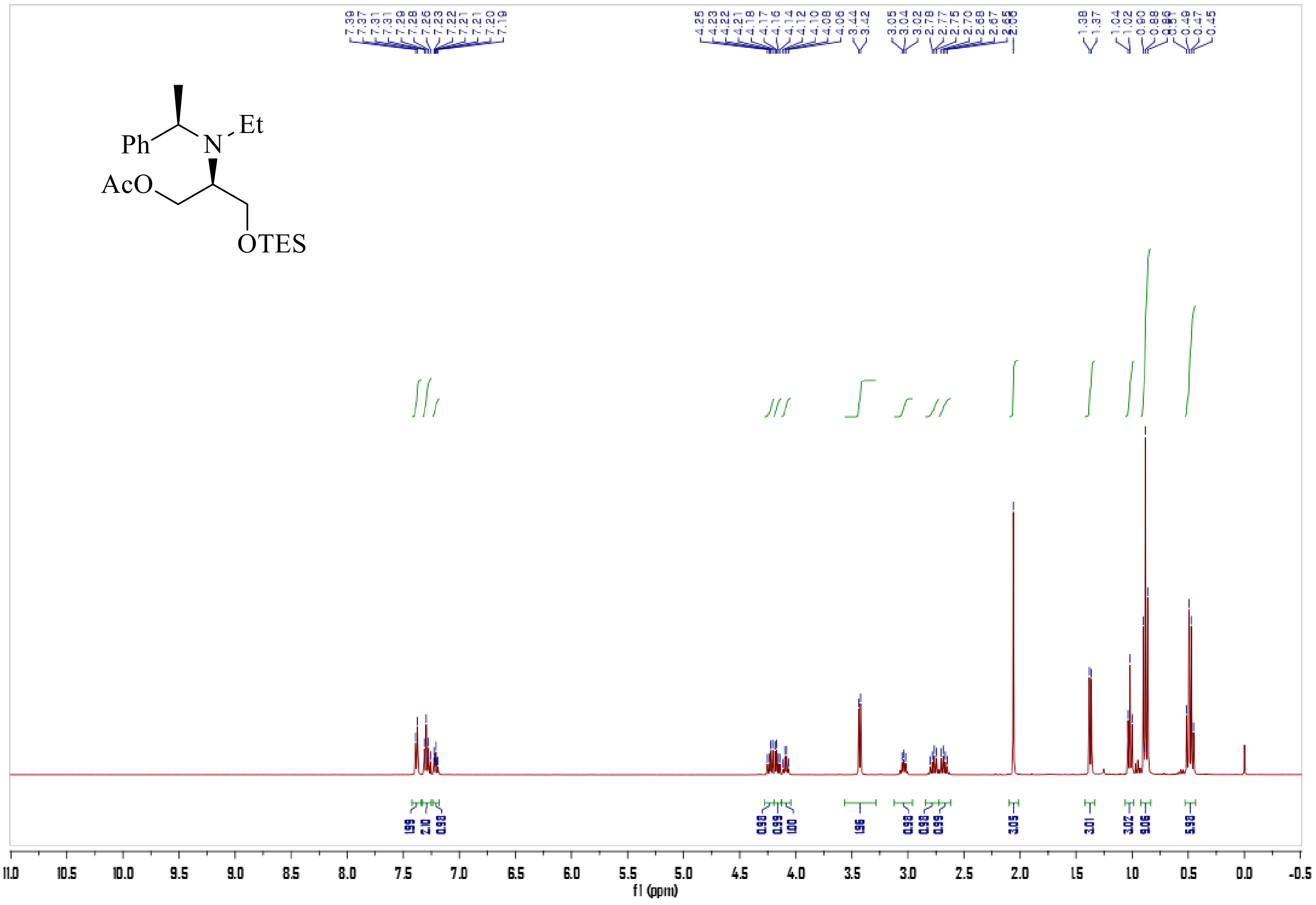

^{13}*C NMR spectrum of (R)-2-(ethyl((R)-1-phenylethyl)amino)-3-((triethylsilyl)oxy)propyl acetate (3BEa):*

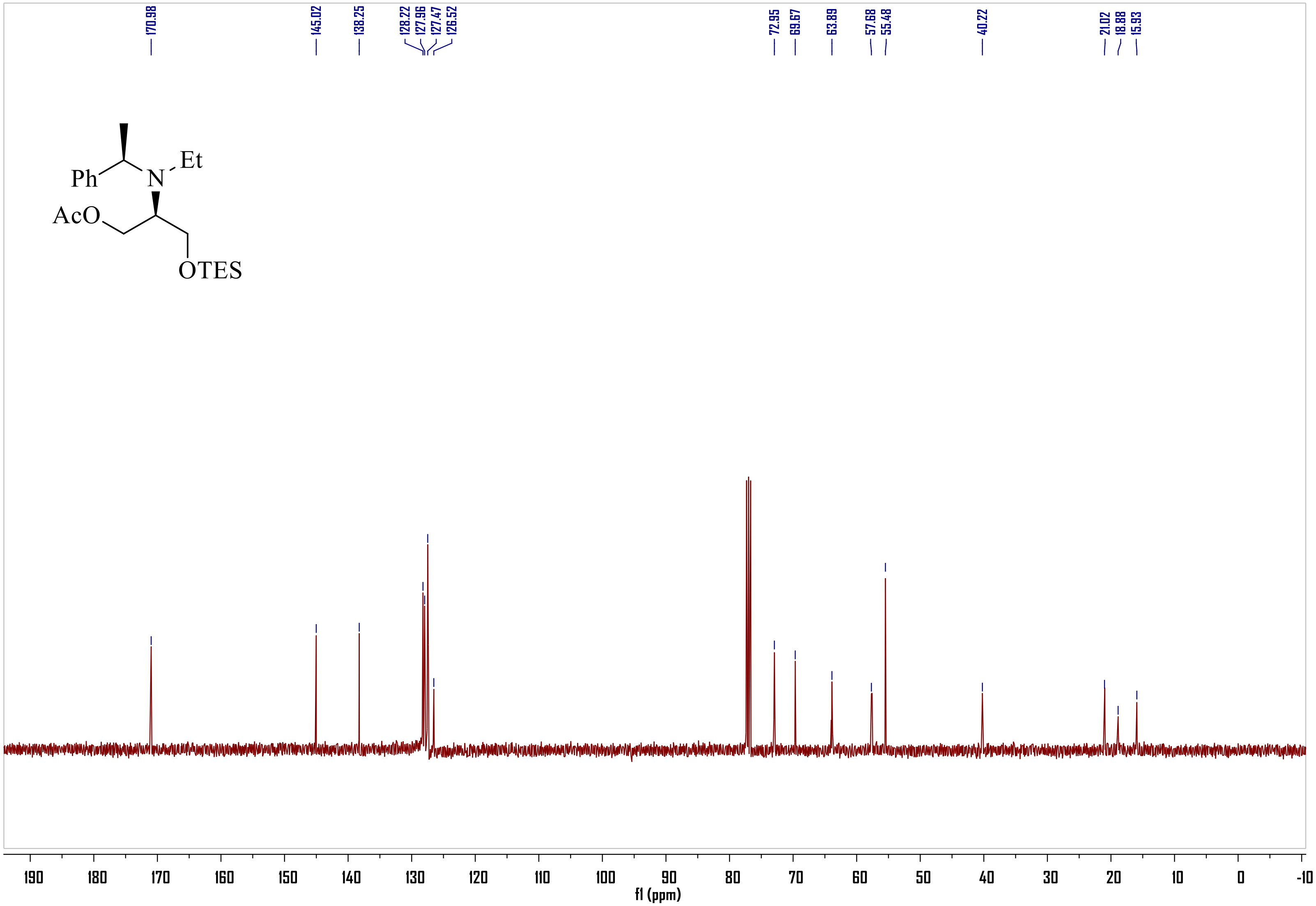

^{1}H NMR spectrum of (S)-1-(ethyl((R)-1-phenylethyl)amino)-3-((triethylsilyl)oxy)propan-2-yl acetate (4BEa):

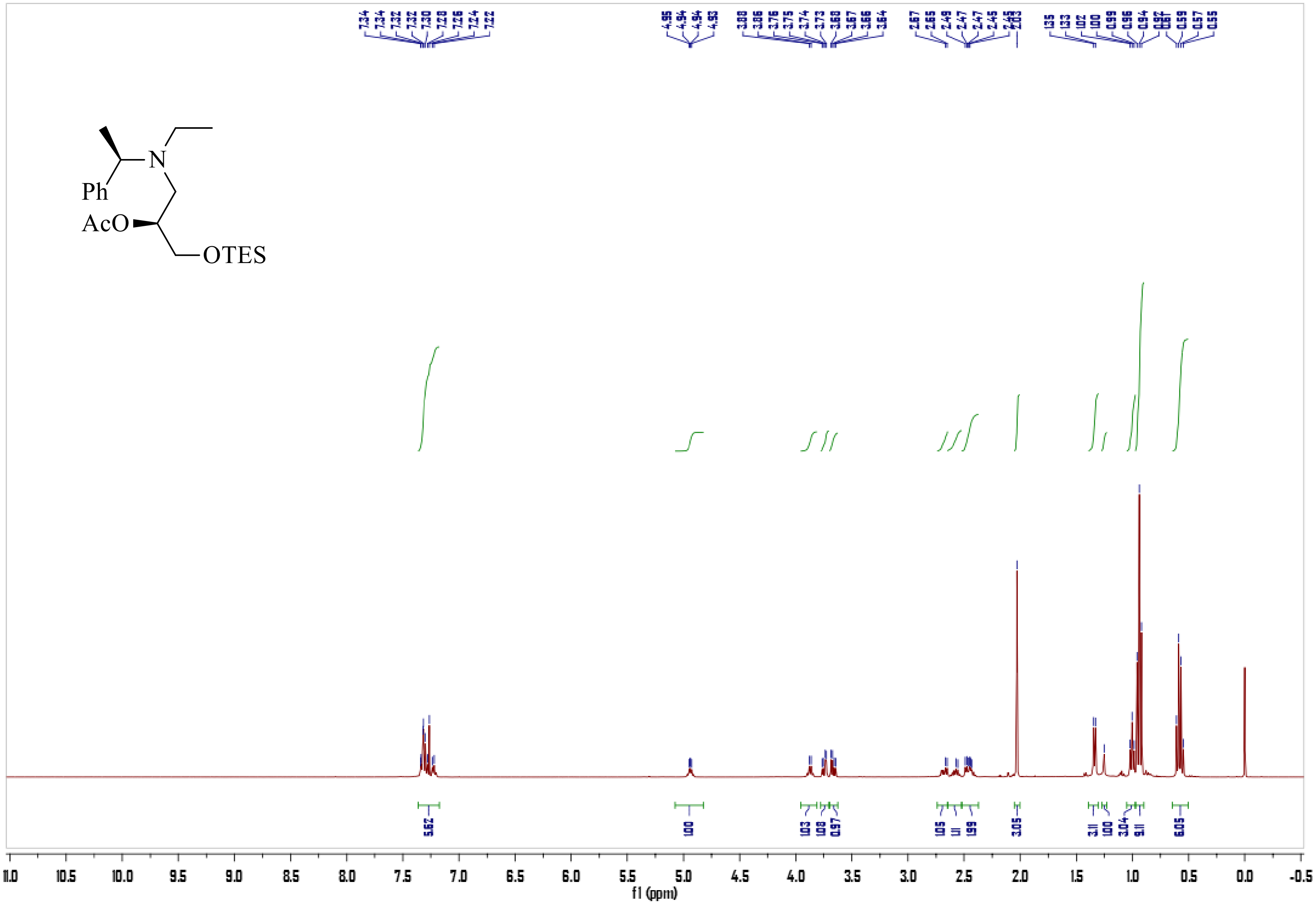

^{13}C NMR spectrum of* (S)-1-(ethyl((R)-1-phenylethyl)amino)-3-((triethylsilyl)oxy)propan-2-yl acetate *(4BEa):

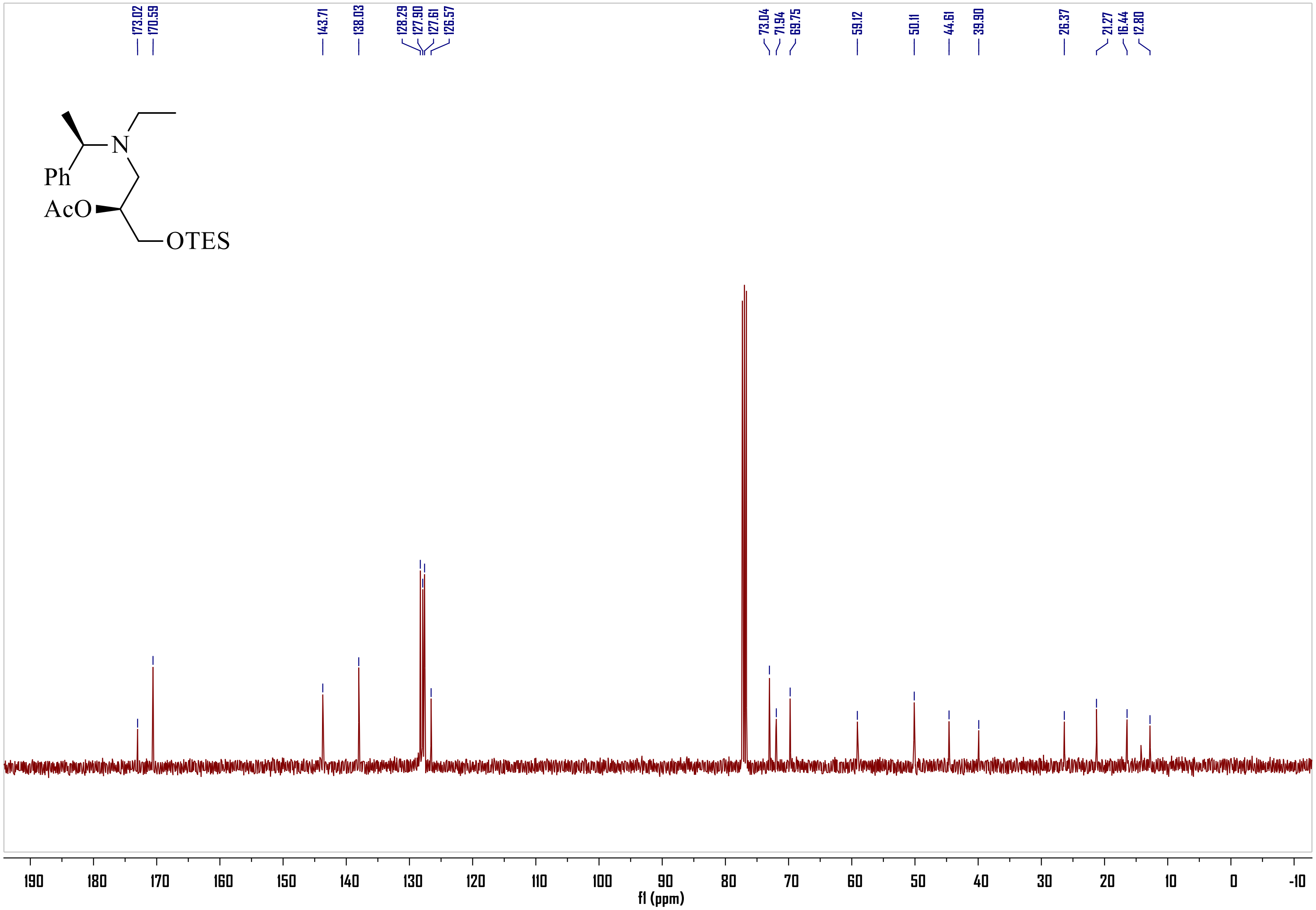

^{1}H NMR spectrum of (S)-2-(((tert-butyldimethylsilyl)oxy)methyl)-1-((R)-1-phenylethyl)aziridine (5):

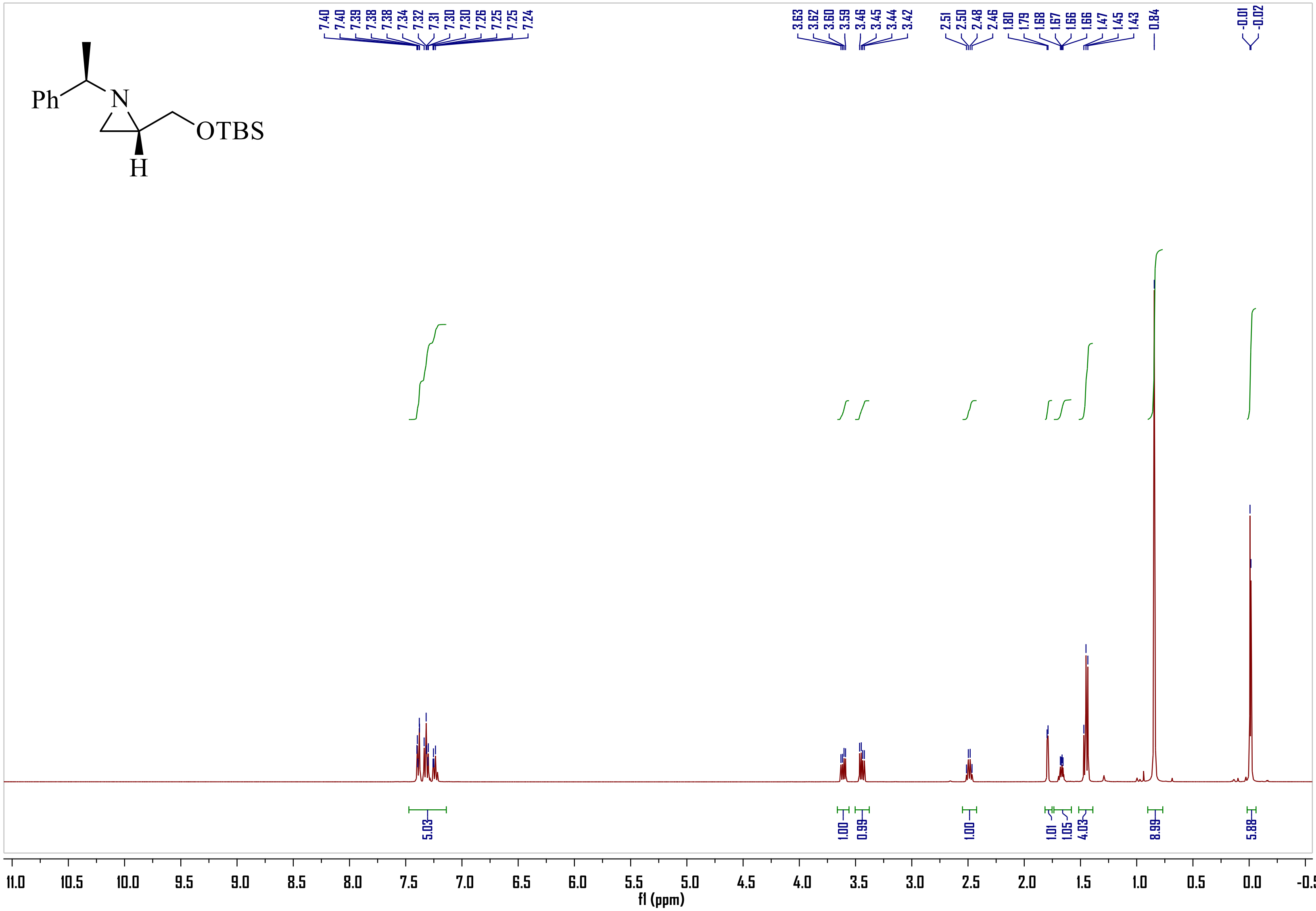

^{13}C NMR spectrum of (S)-2-(((tert-butyldimethylsilyl)oxy)methyl)-1-((R)-1-phenylethyl)aziridine (5):

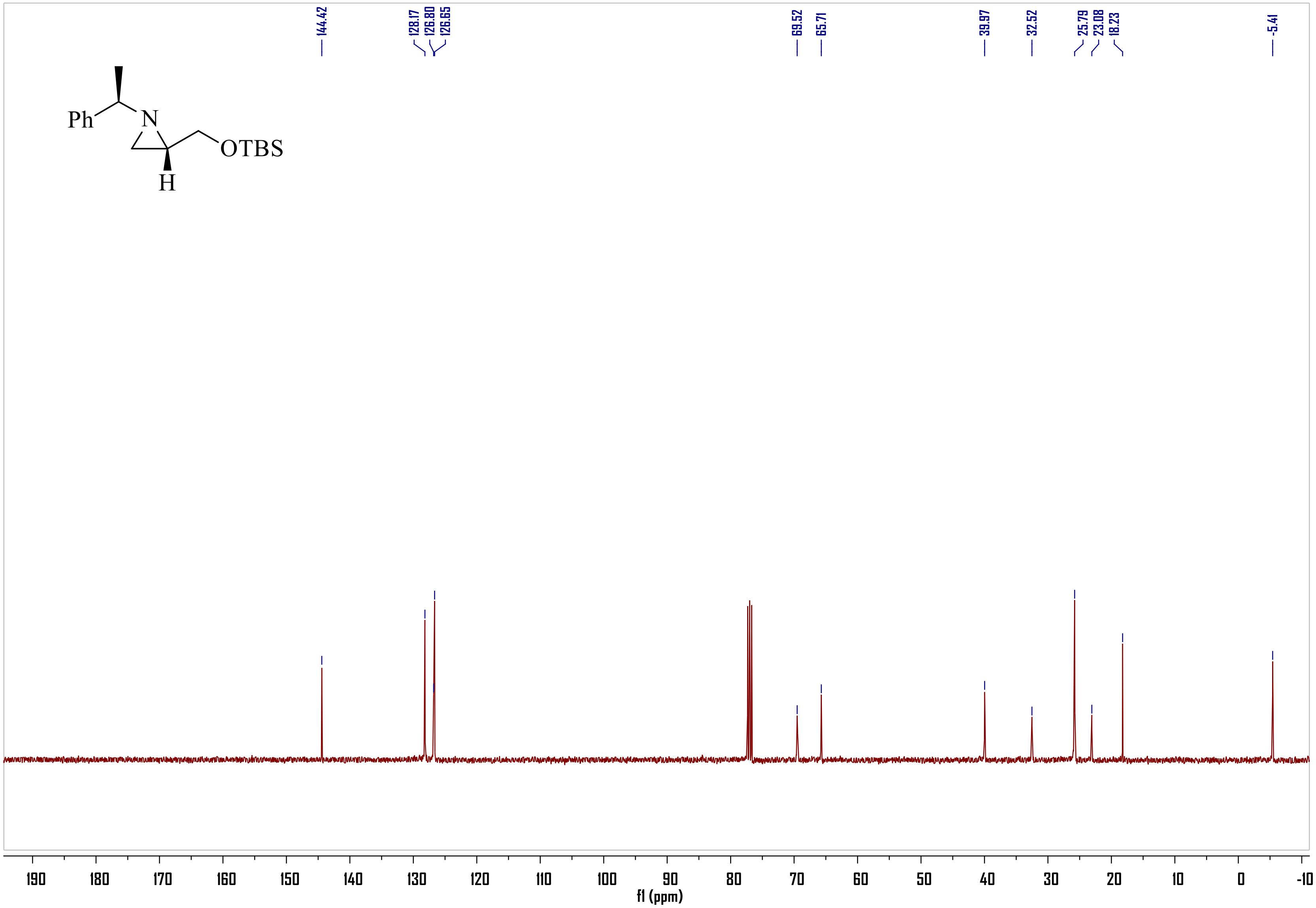

^{1}H NMR spectrum of (S)-3-((tert-butyldimethylsilyl)oxy)-2-(ethyl((R)-1-phenylethyl)amino)propyl acetate (6):

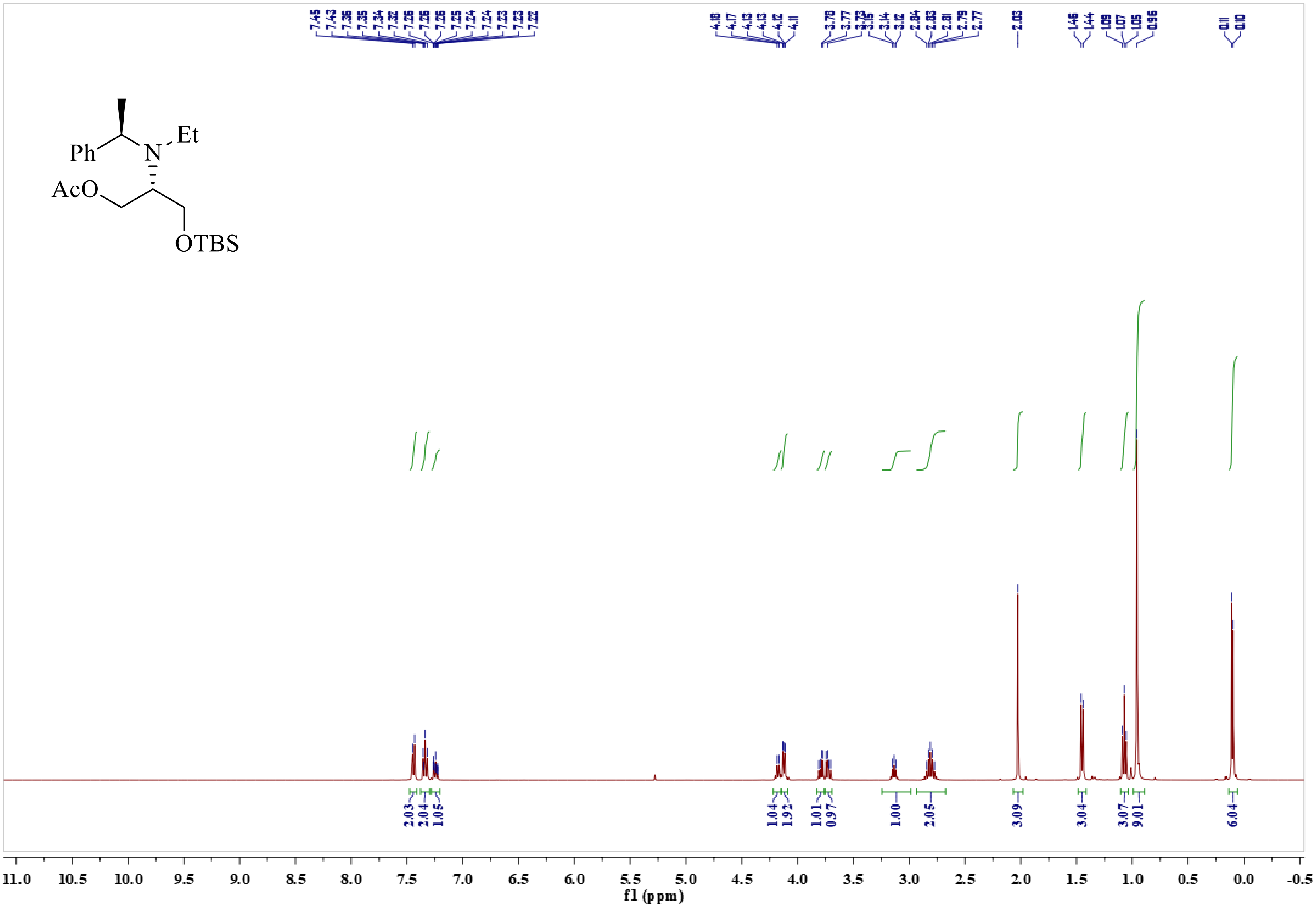

^{13}C NMR spectrum of (S)-3-((tert-butyldimethylsilyl)oxy)-2-(ethyl((R)-1-phenylethyl)amino)propyl acetate (6):

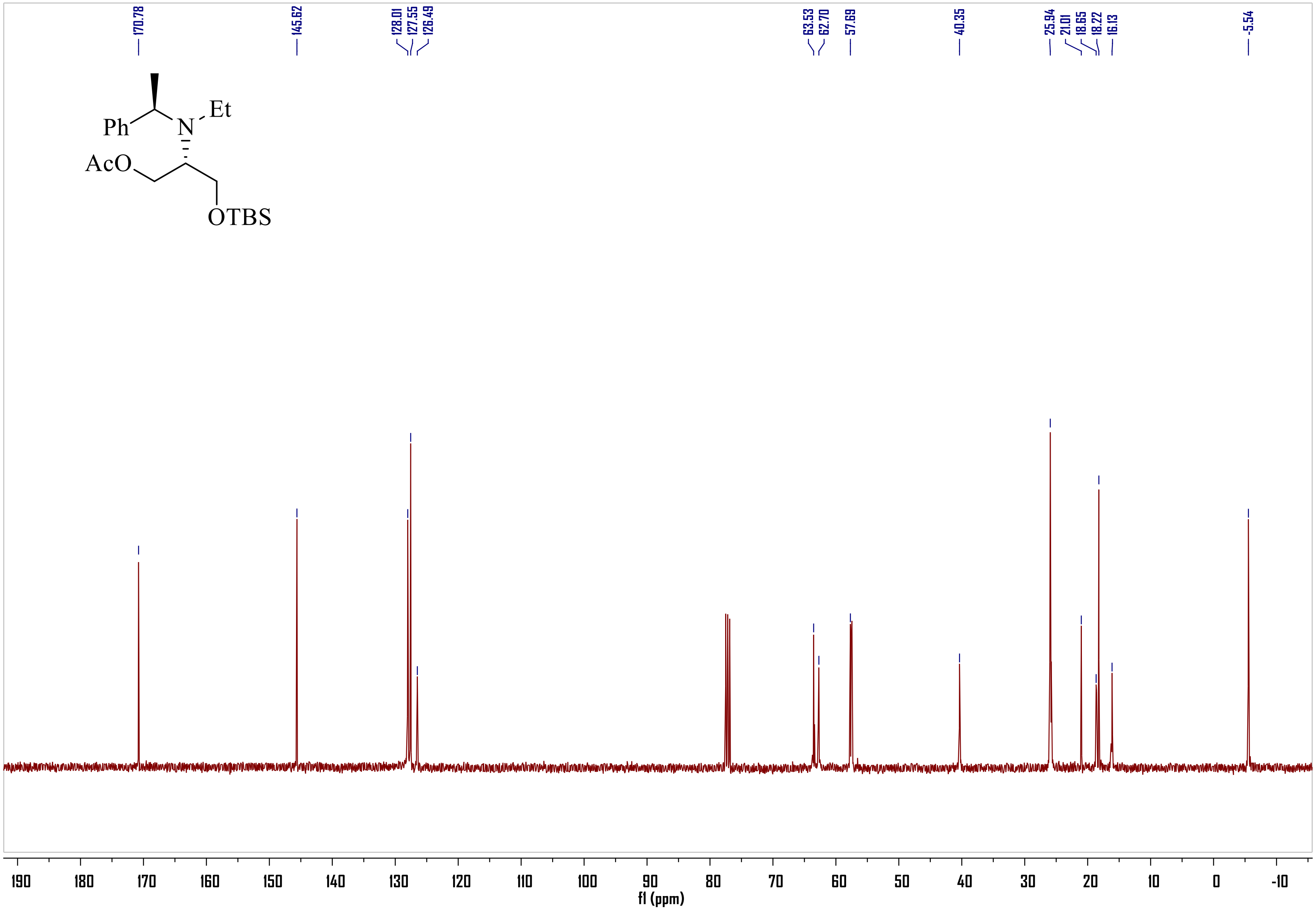

^{1}H NMR spectrum of (S)-3-((tert-butyldimethylsilyl)oxy)-2-(ethyl((R)-1-phenylethyl)amino)propan-1-ol (7):

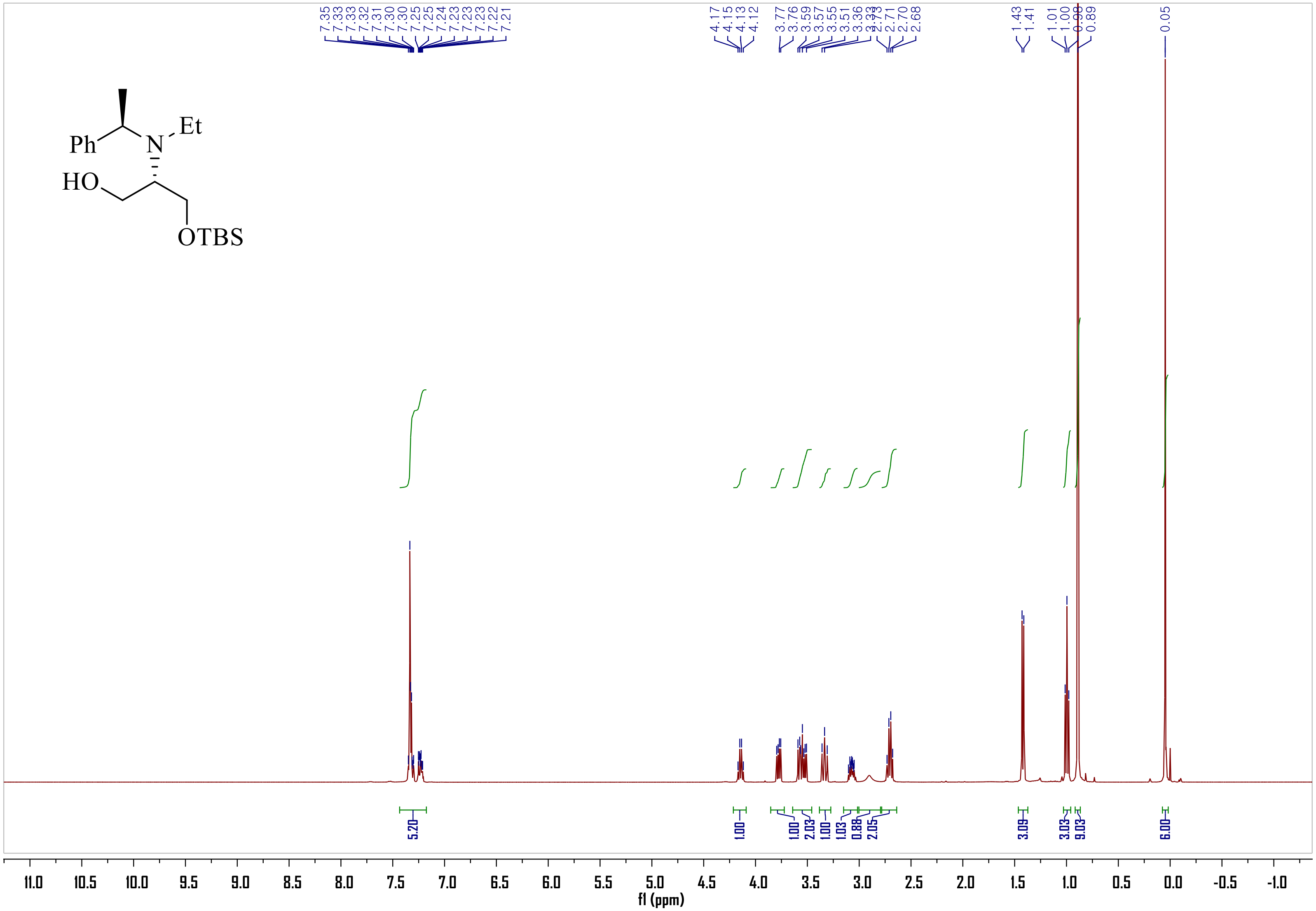

^{13}C NMR spectrum of (S)-3-((tert-butyldimethylsilyl)oxy)-2-(ethyl((R)-1-phenylethyl)amino)propan-1-ol (7):

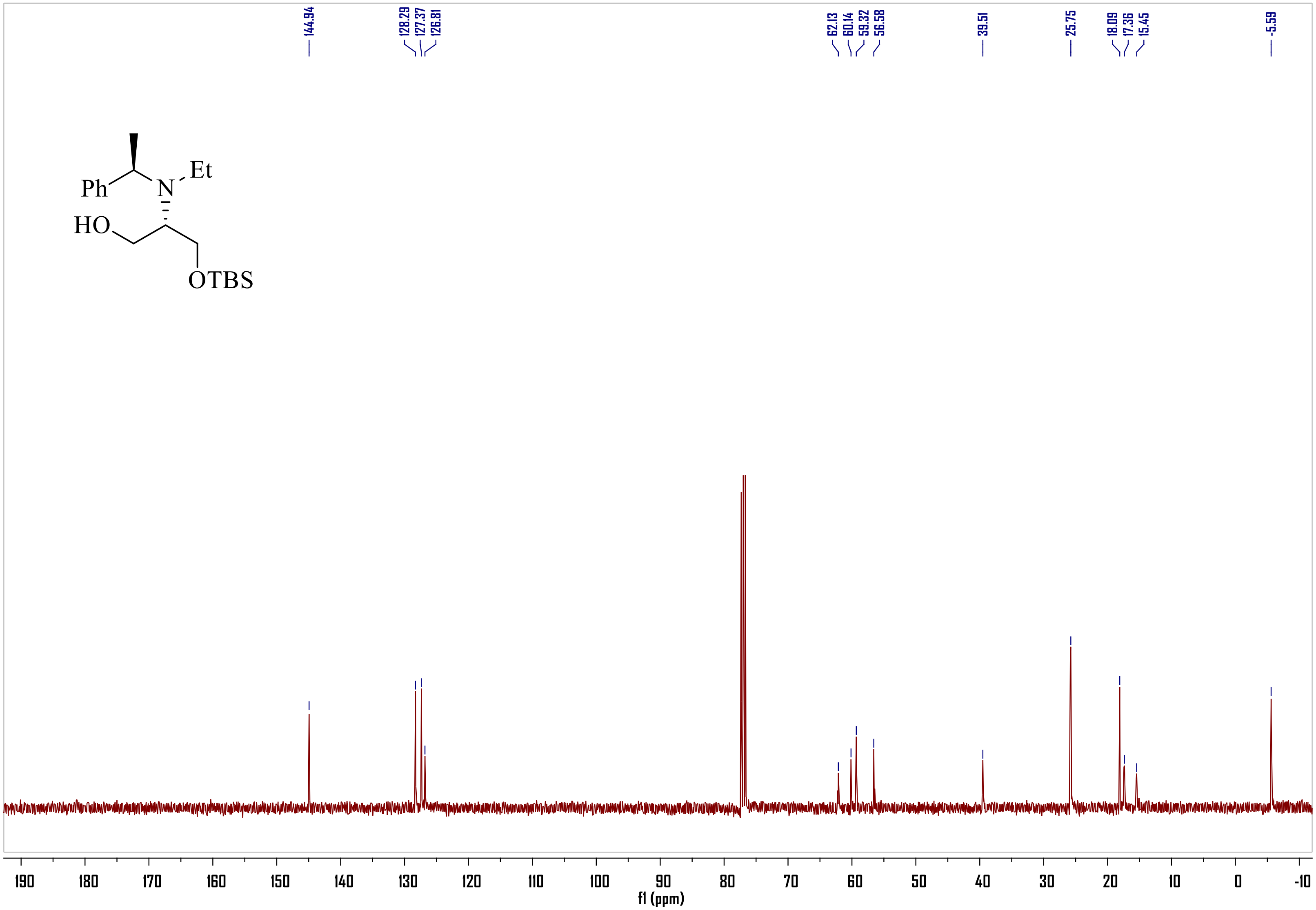

^{1}H NMR spectrum of (S)-tert-butyl (1-((tert-butyldimethylsilyl)oxy)-3-hydroxypropan-2-yl)(ethyl)carbamate (8):

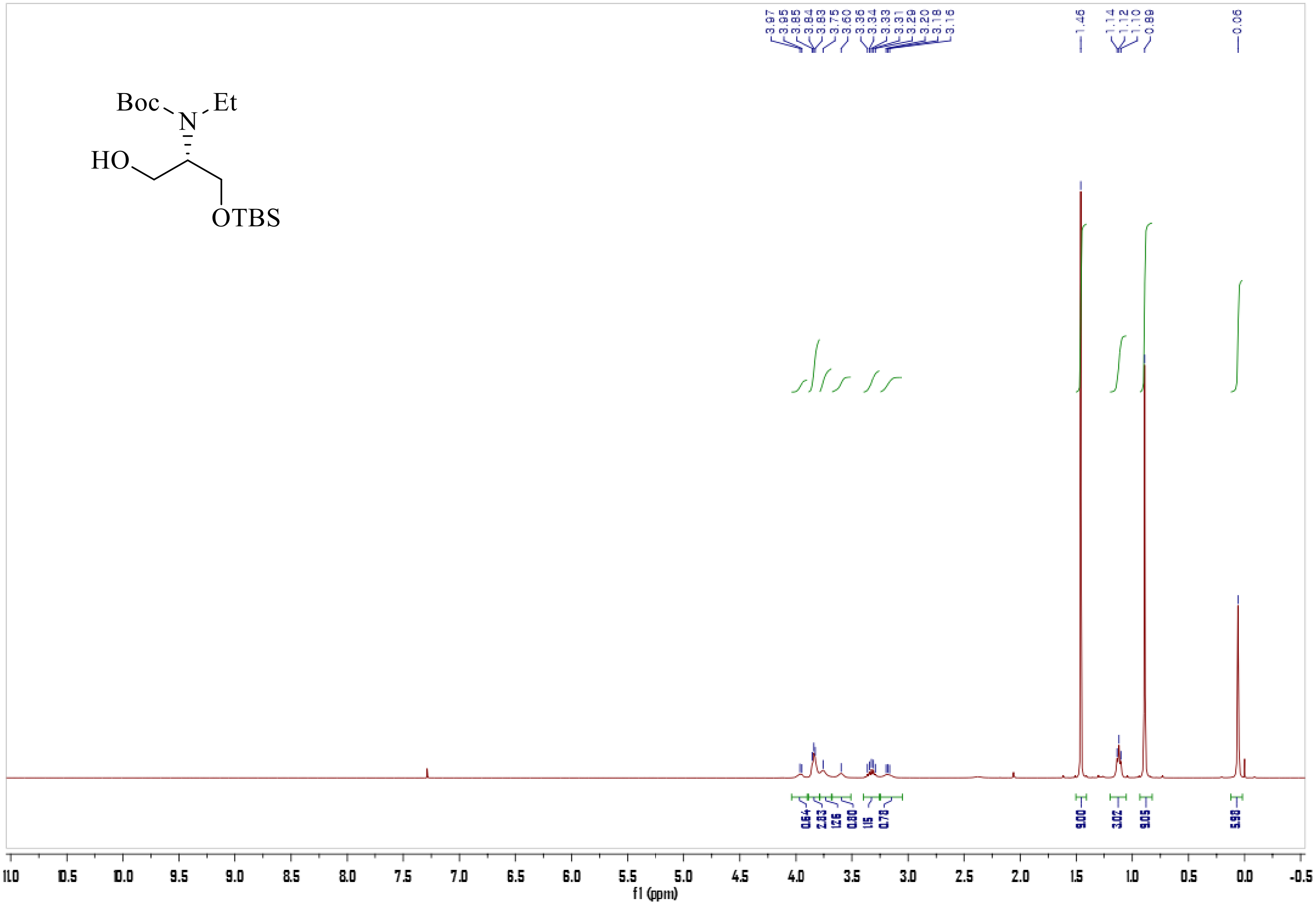

^{13}C NMR spectrum of (S)-tert-butyl (1-((tert-butyldimethylsilyl)oxy)-3-hydroxypropan-2-yl)(ethyl)carbamate (8):

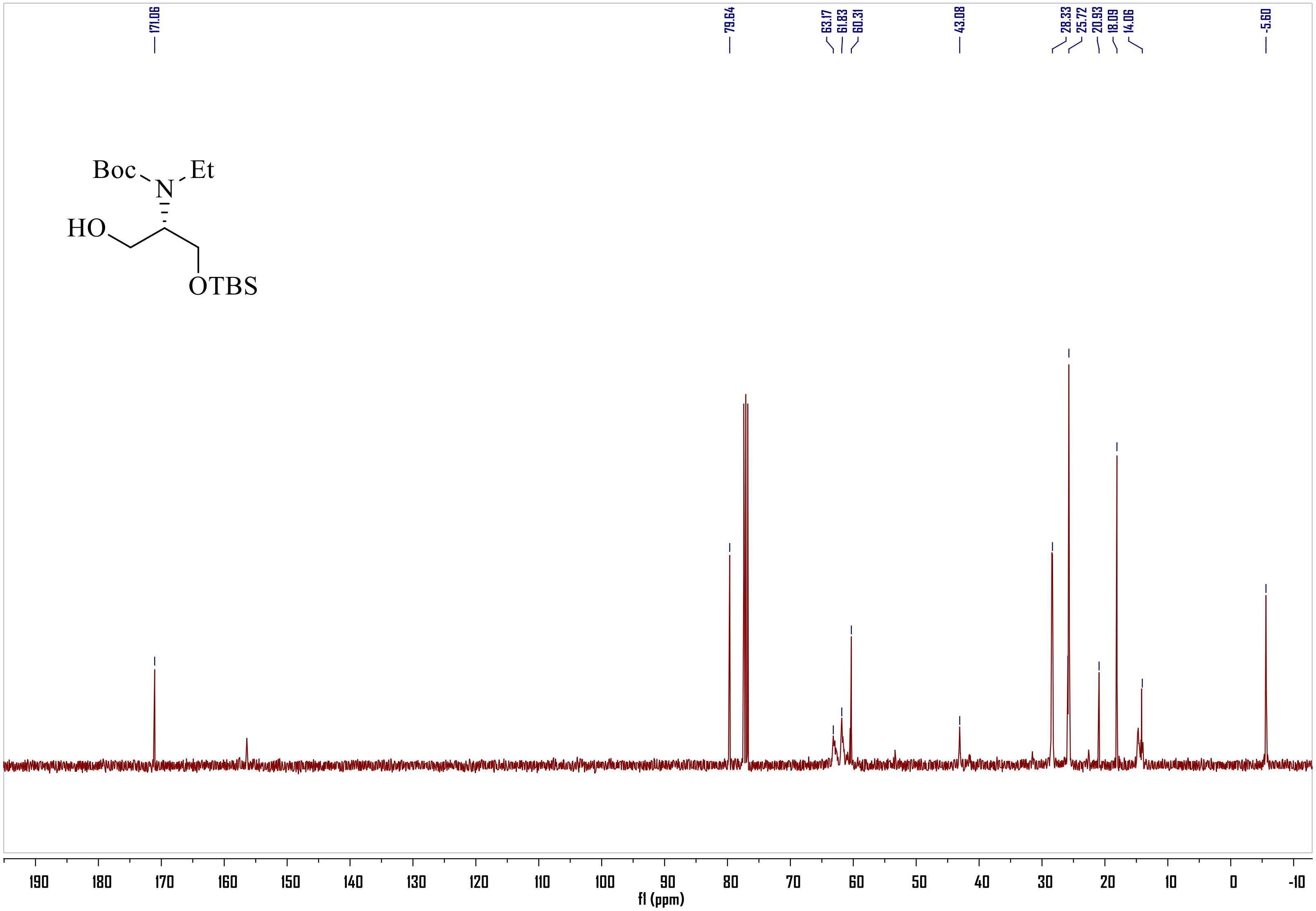

^{1}H NMR spectrum of (S)-4-(((tert-butyldimethylsilyl)oxy)methyl)-3-ethyloxazolidin-2-one (9):

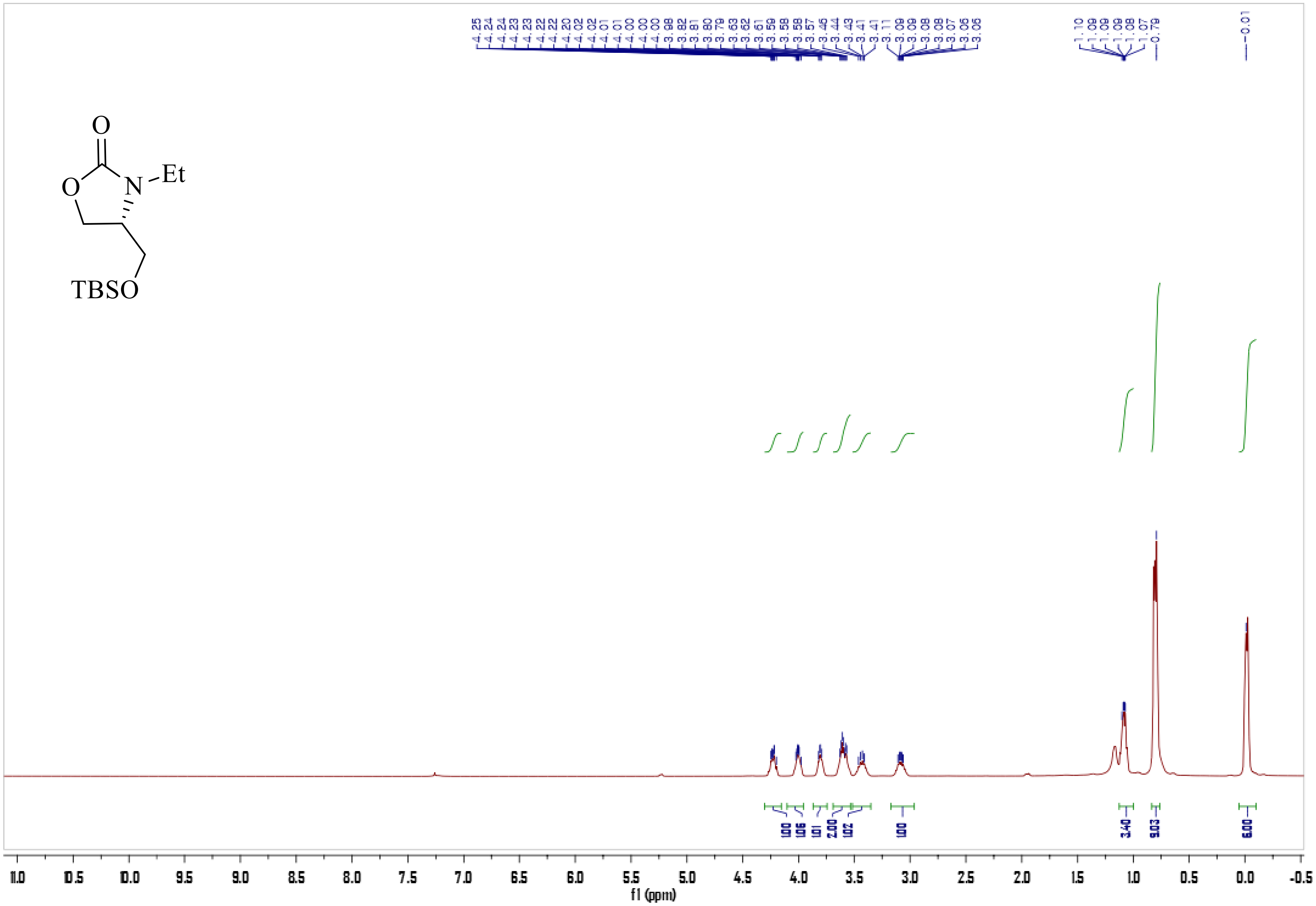

^{13}C NMR spectrum of (S)-4-(((tert-butyldimethylsilyl)oxy)methyl)-3-ethyloxazolidin-2-one (9):

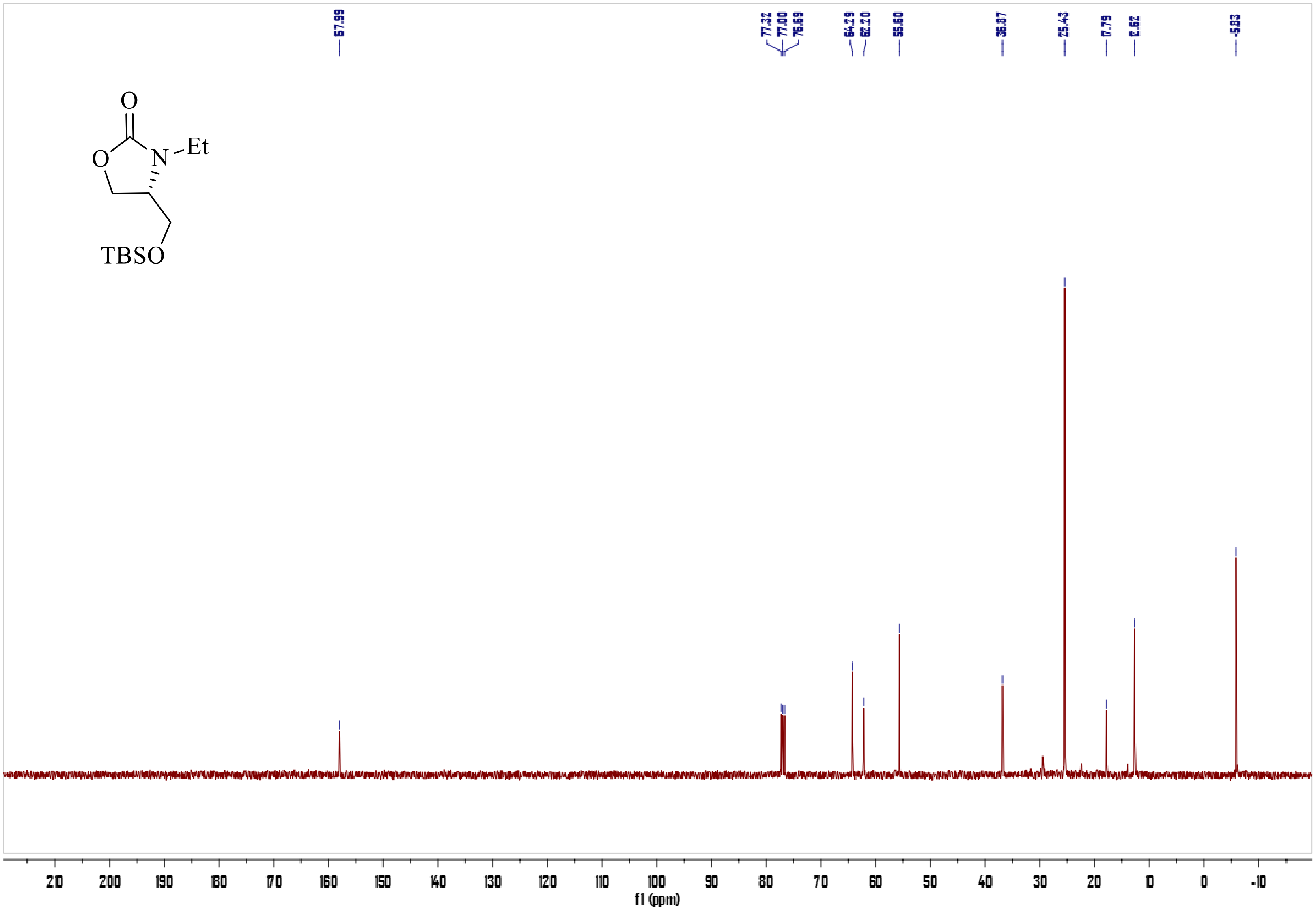

^{1}H NMR spectrum of (R)-3-ethyl-4-(hydroxymethyl)oxazolidin-2-one (9 ’):

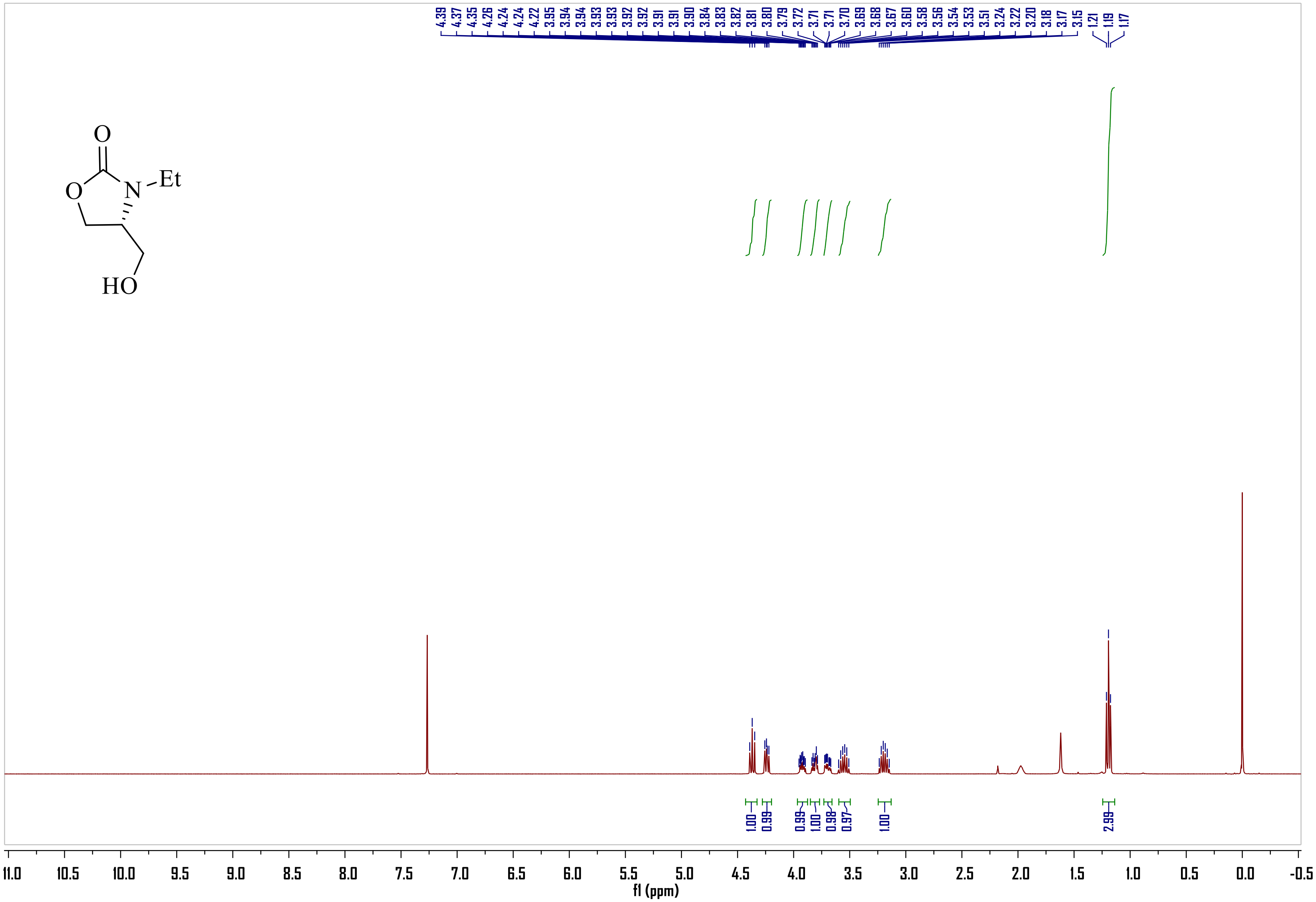

^{13}C NMR spectrum of (R)-3-ethyl-4-(hydroxymethyl)oxazolidin-2-one (9 ’):

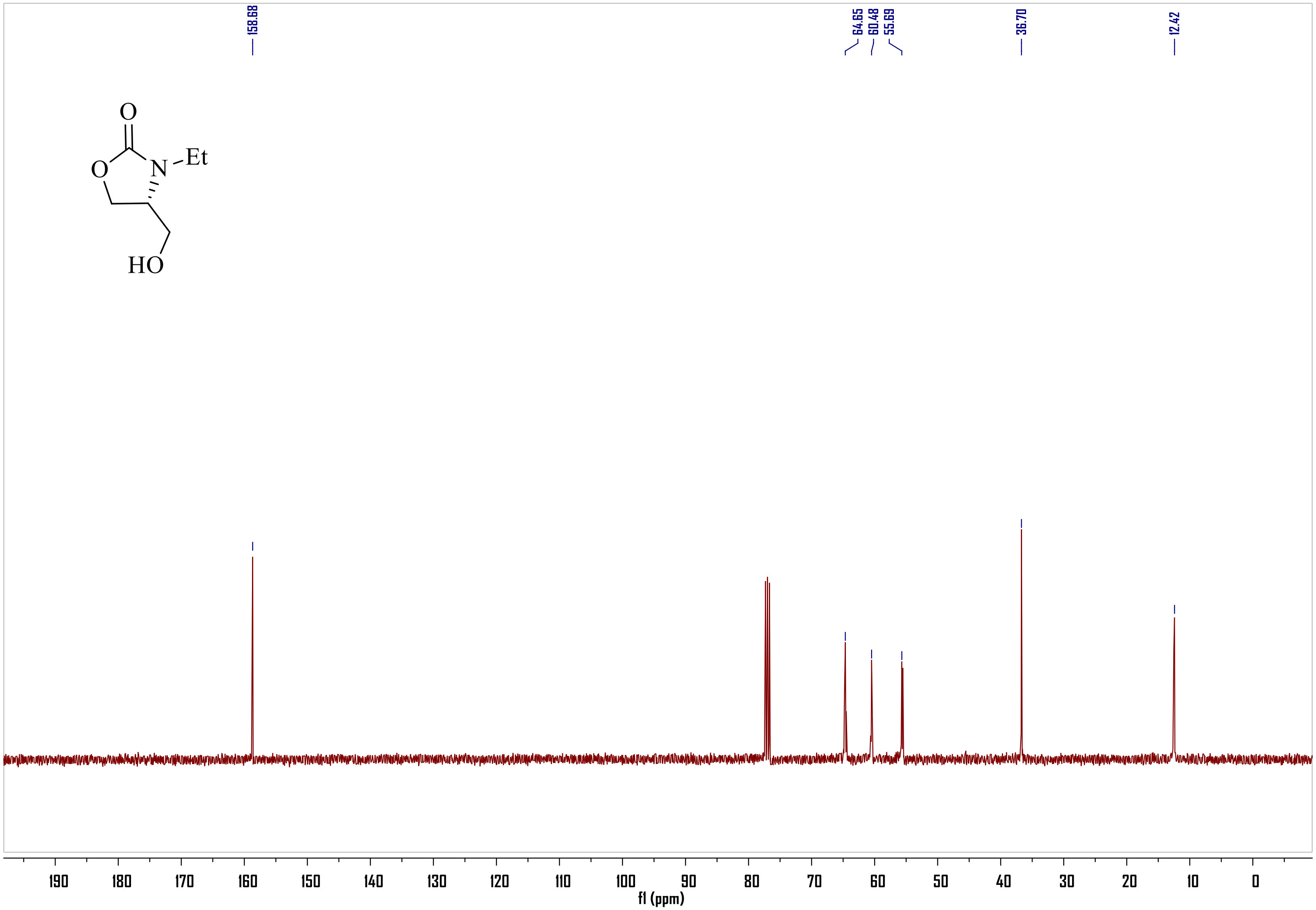

^{1}H NMR spectrum of (S)-3-ethyl-2-oxooxazolidine-4-carbaldehyde (10):

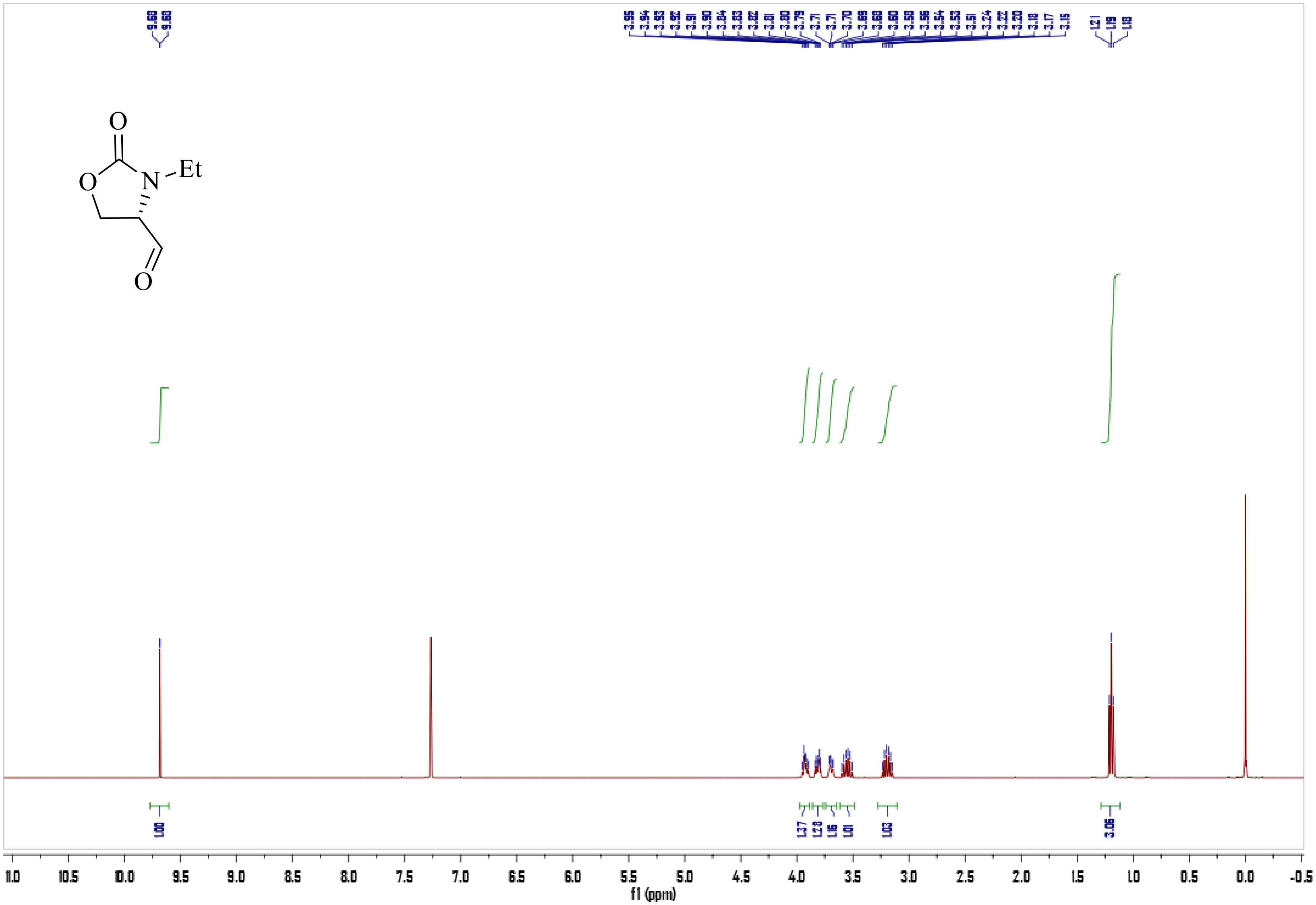

13C NMR spectrum of (S)-3-ethyl-2-oxooxazolidine-4-carbaldehyde (10):

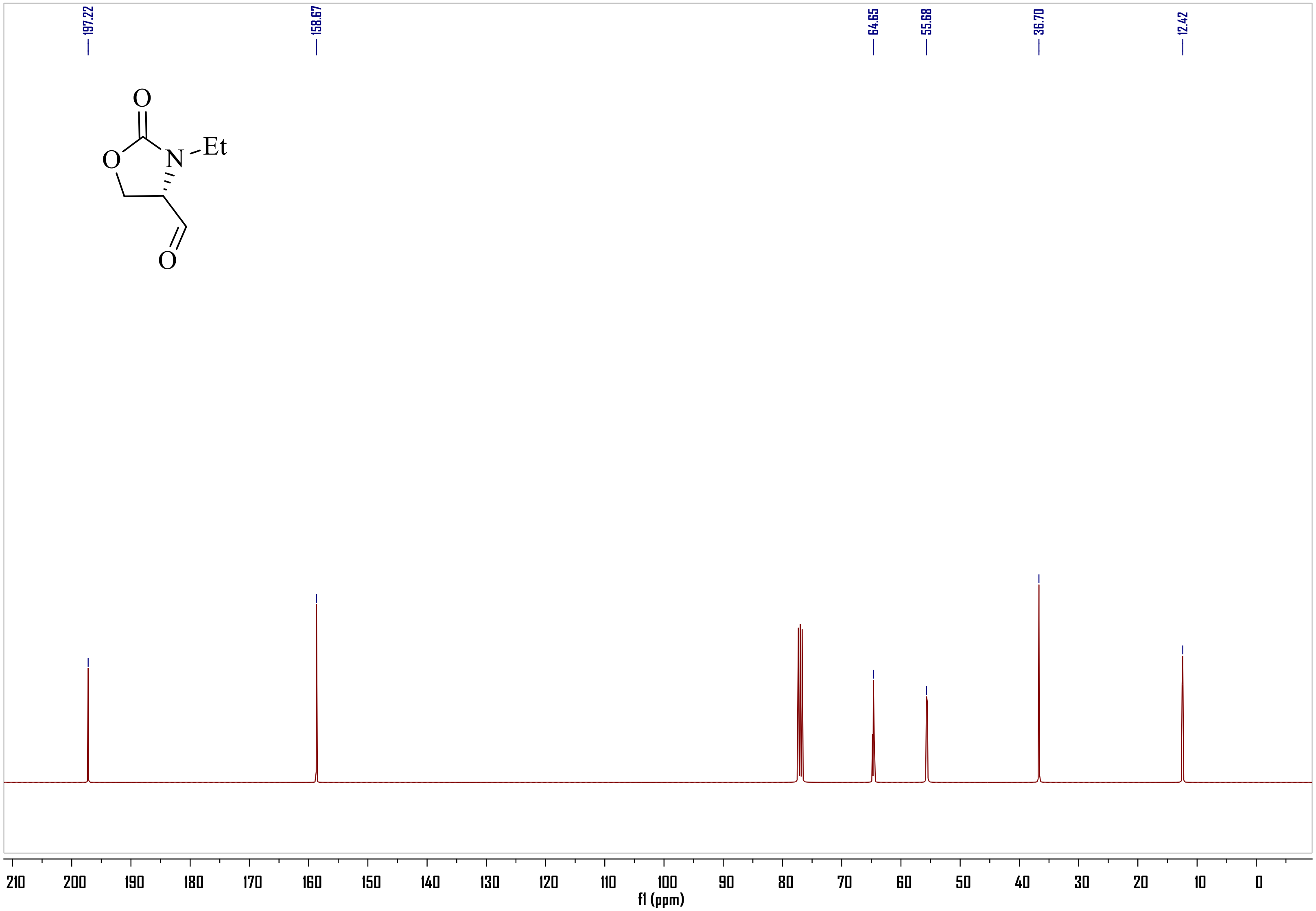

^{1}H NMR spectrum of (S)-2-(allyl((R)-1-phenylethyl)amino)-3-(benzyloxy)propyl acetate (3AAa):

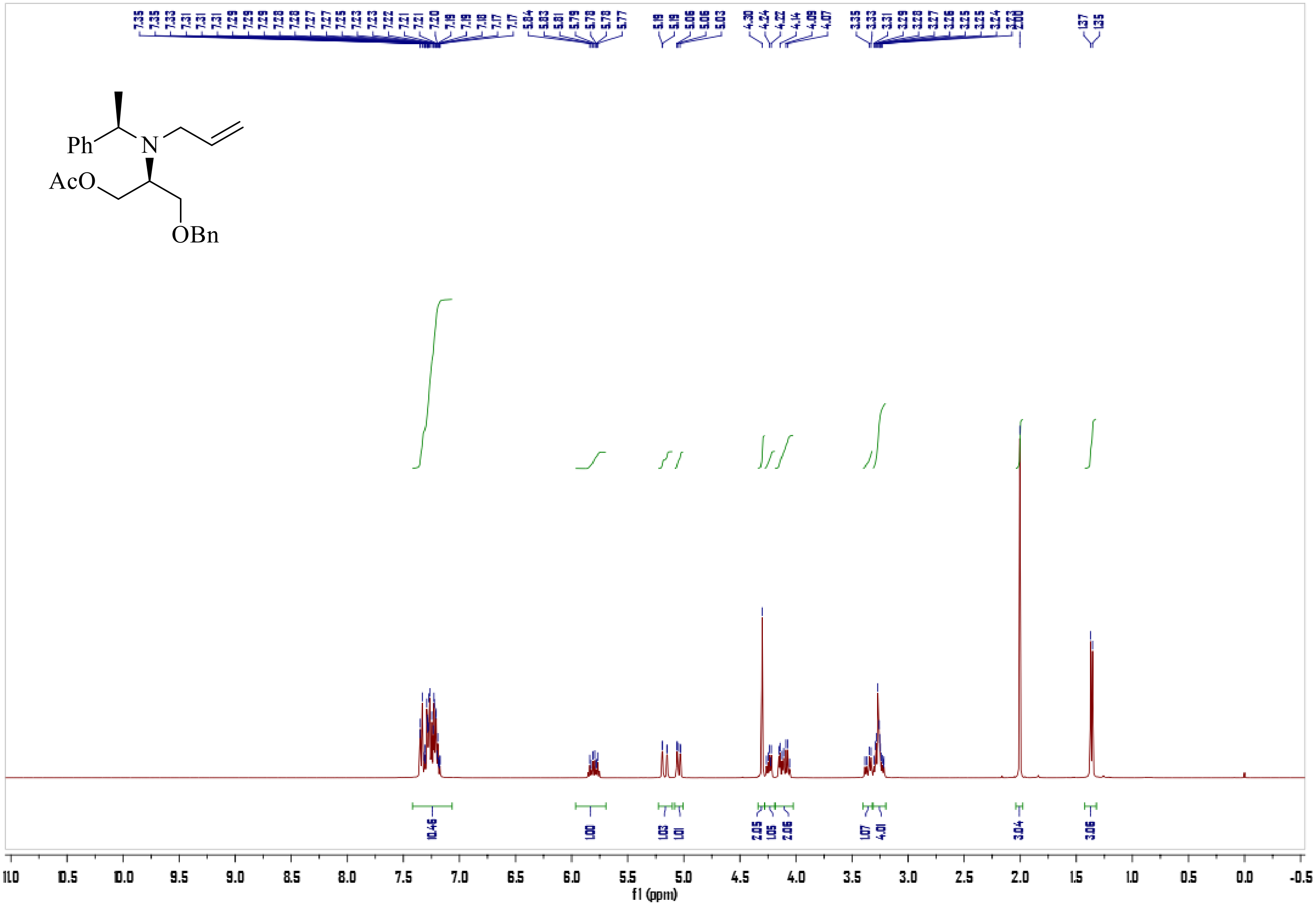

^{13}C NMR spectrum of (S)-2-(allyl((R)-1-phenylethyl)amino)-3-(benzyloxy)propyl acetate (3AAa):

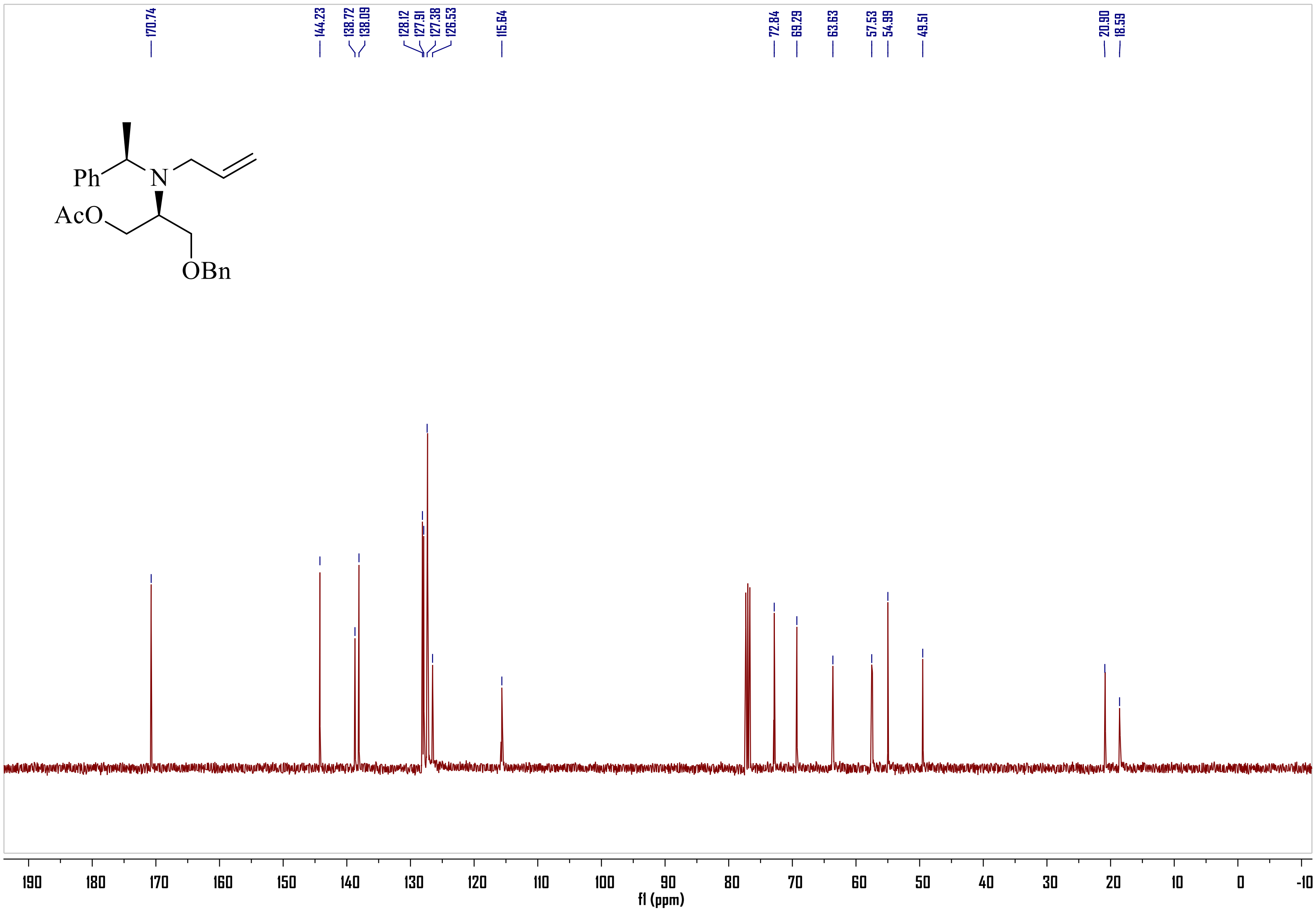

^{1}H NMR spectrum of (S)-2-(allyl((R)-1-phenylethyl)amino)-3-((tert-butyldimethylsilyl)oxy)propyl acetate (3CAa):

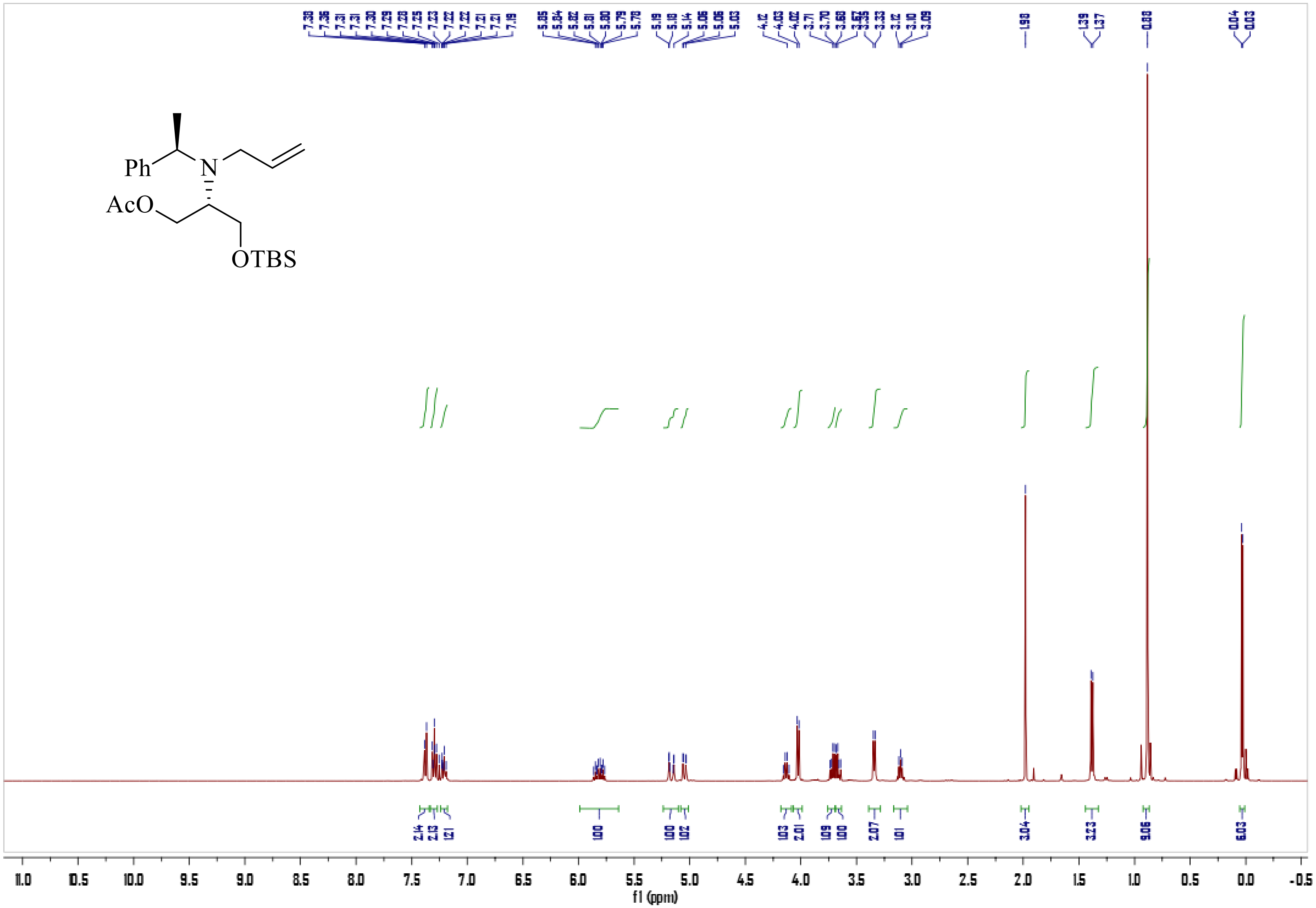

^{13}C NMR spectrum of (S)-2-(allyl((R)-1-phenylethyl)amino)-3-((tert-butyldimethylsilyl)oxy)propyl acetate (3CAa):

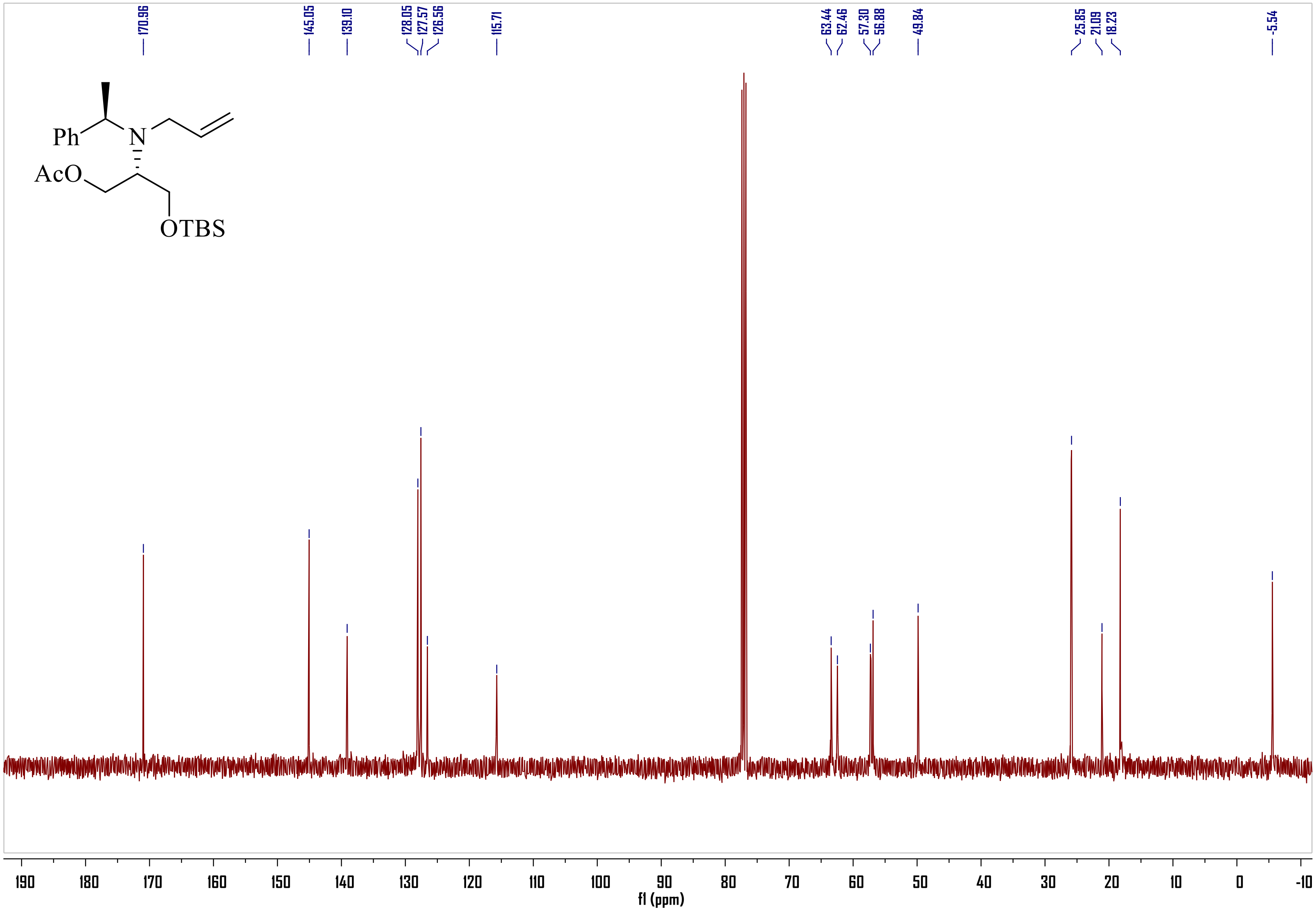

¹H NMR spectrum of (R)-ethyl 3-acetoxy-2-(allyl((R)-1-phenylethyl)amino)propanoate (3DAa):

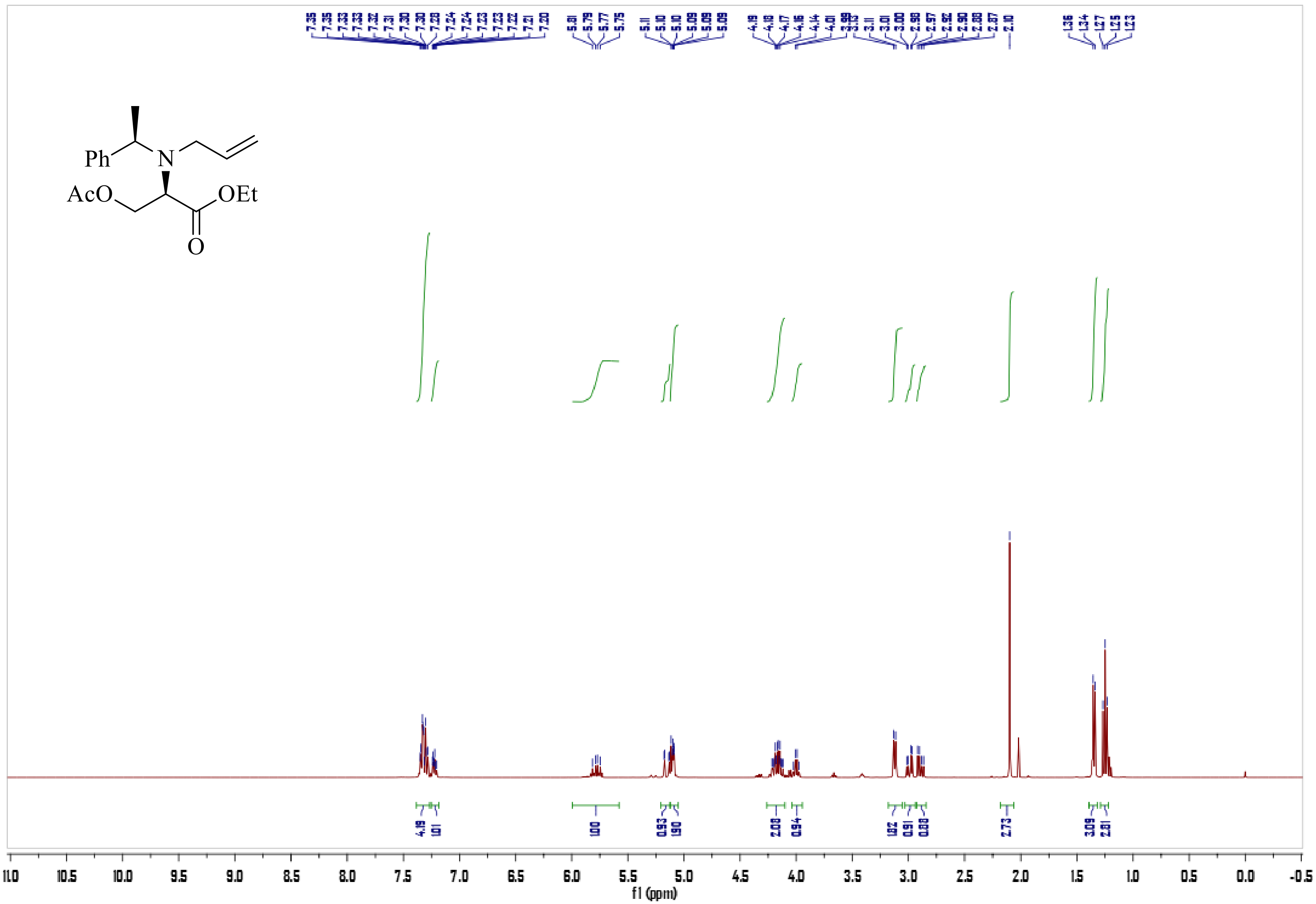

^{13}C NMR spectrum of (R)-ethyl 3-acetoxy-2-(allyl((R)-1-phenylethyl)amino)propanoate (3DAa):

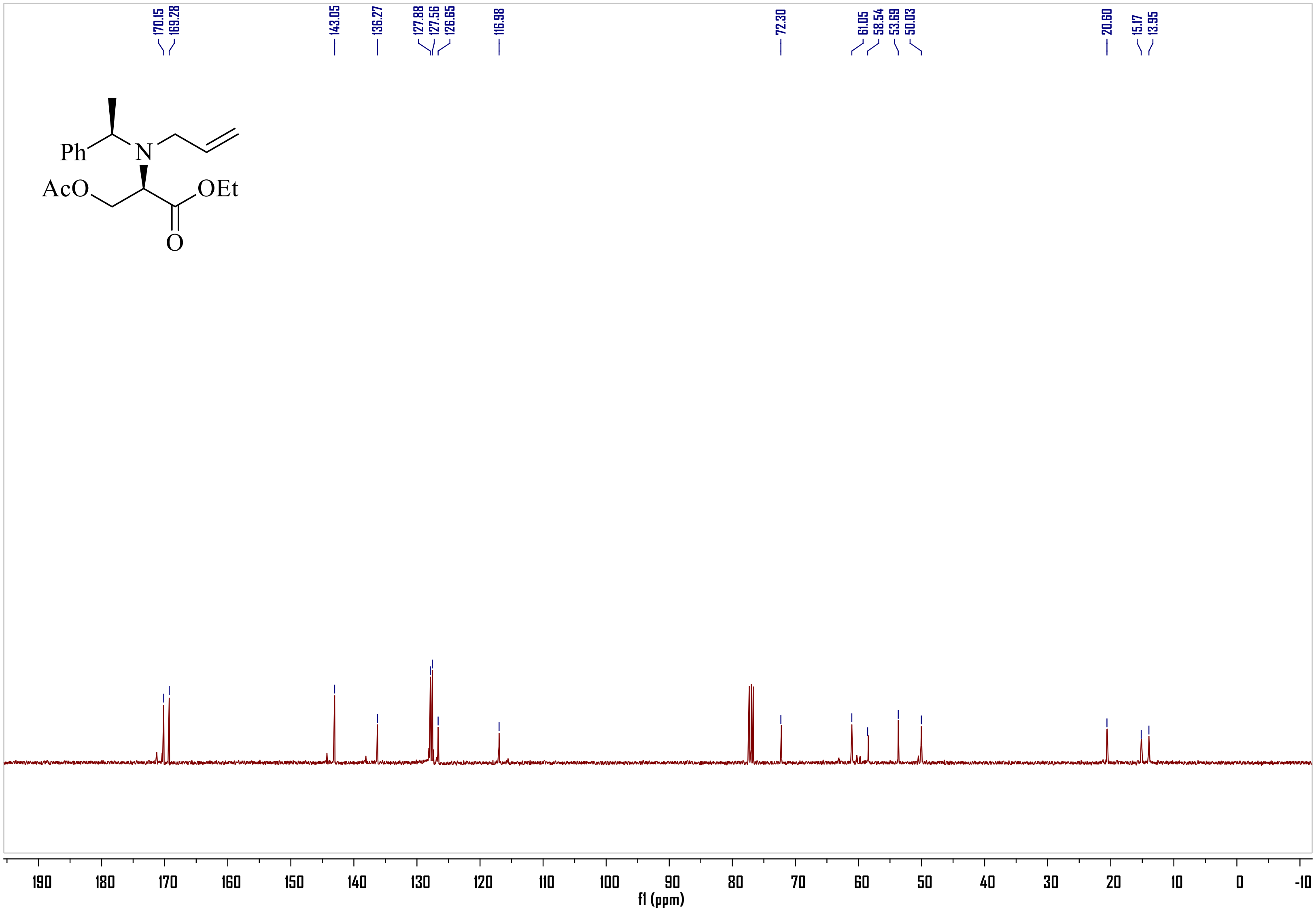

^{1}H NMR spectrum of (R)-ethyl 2-(allyl((R)-1-phenylethyl)amino)-3-azidopropanoate (3DAb):

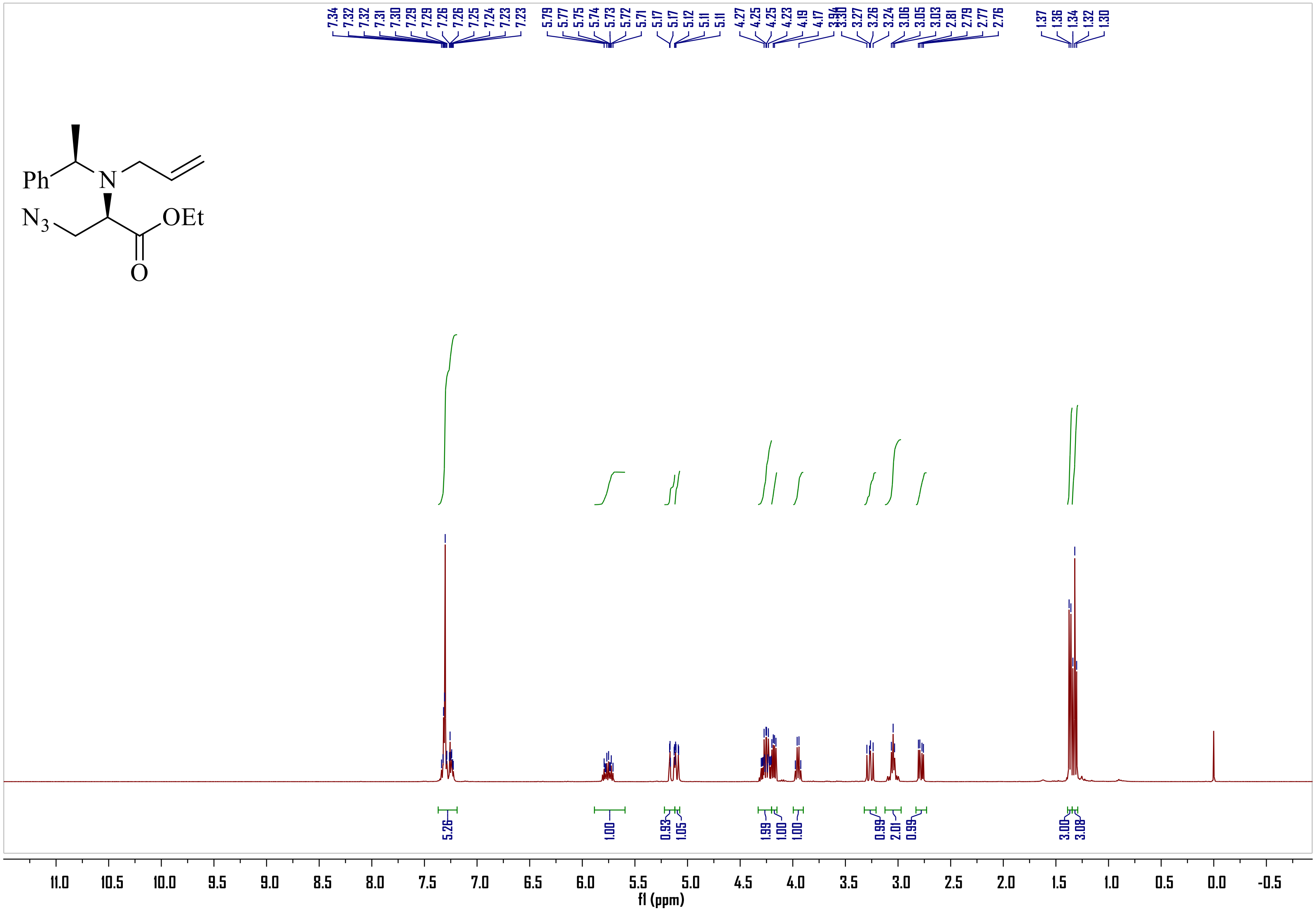

^{13}C NMR spectrum of (R)-ethyl 2-(allyl((R)-1-phenylethyl)amino)-3-azidopropanoate (3DAb):

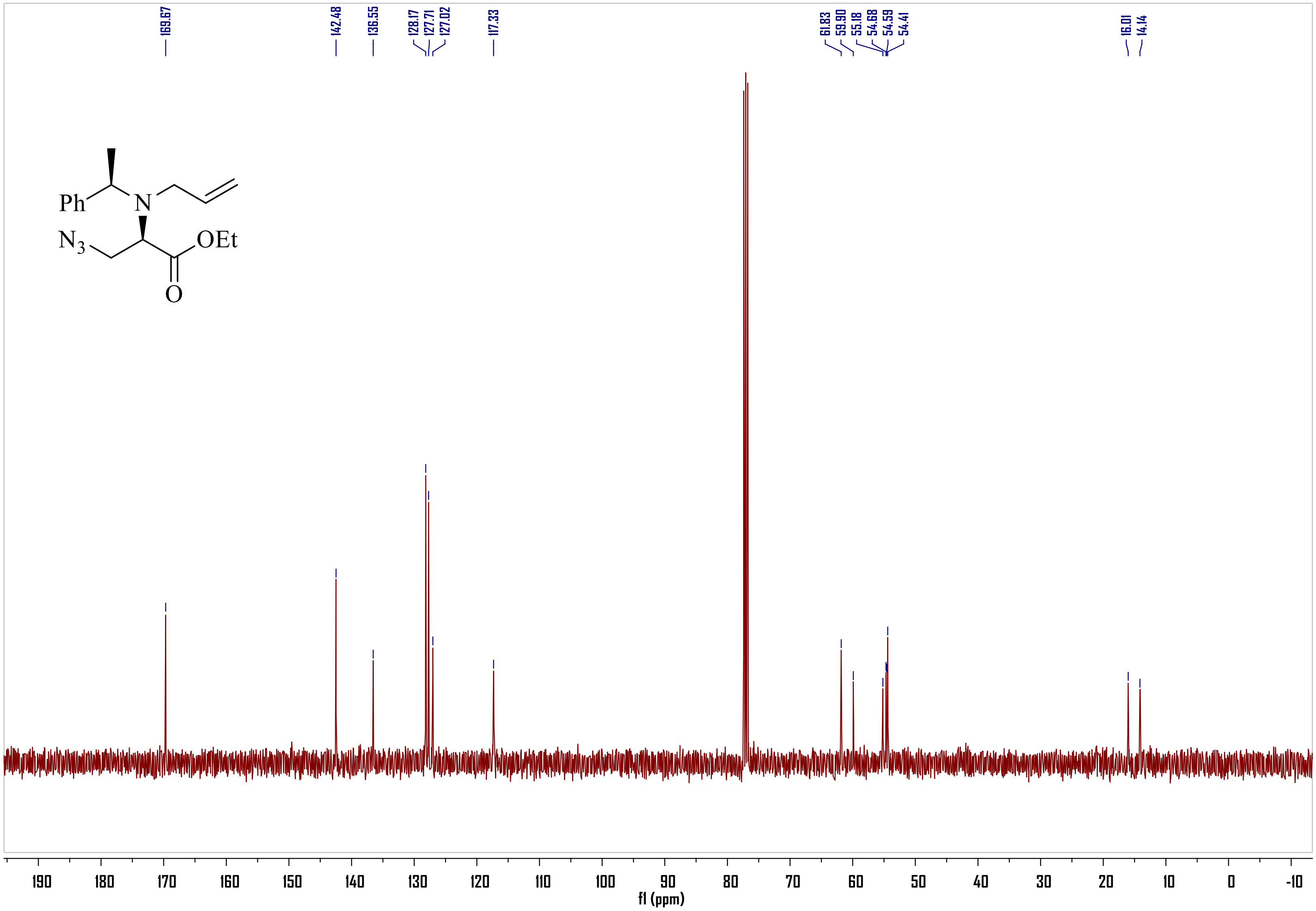

^{1}H NMR spectrum (R)-2-(allyl((R)-1-phenylethyl)amino)-3-(allyloxy)-3-methylbutyl acetate (12):

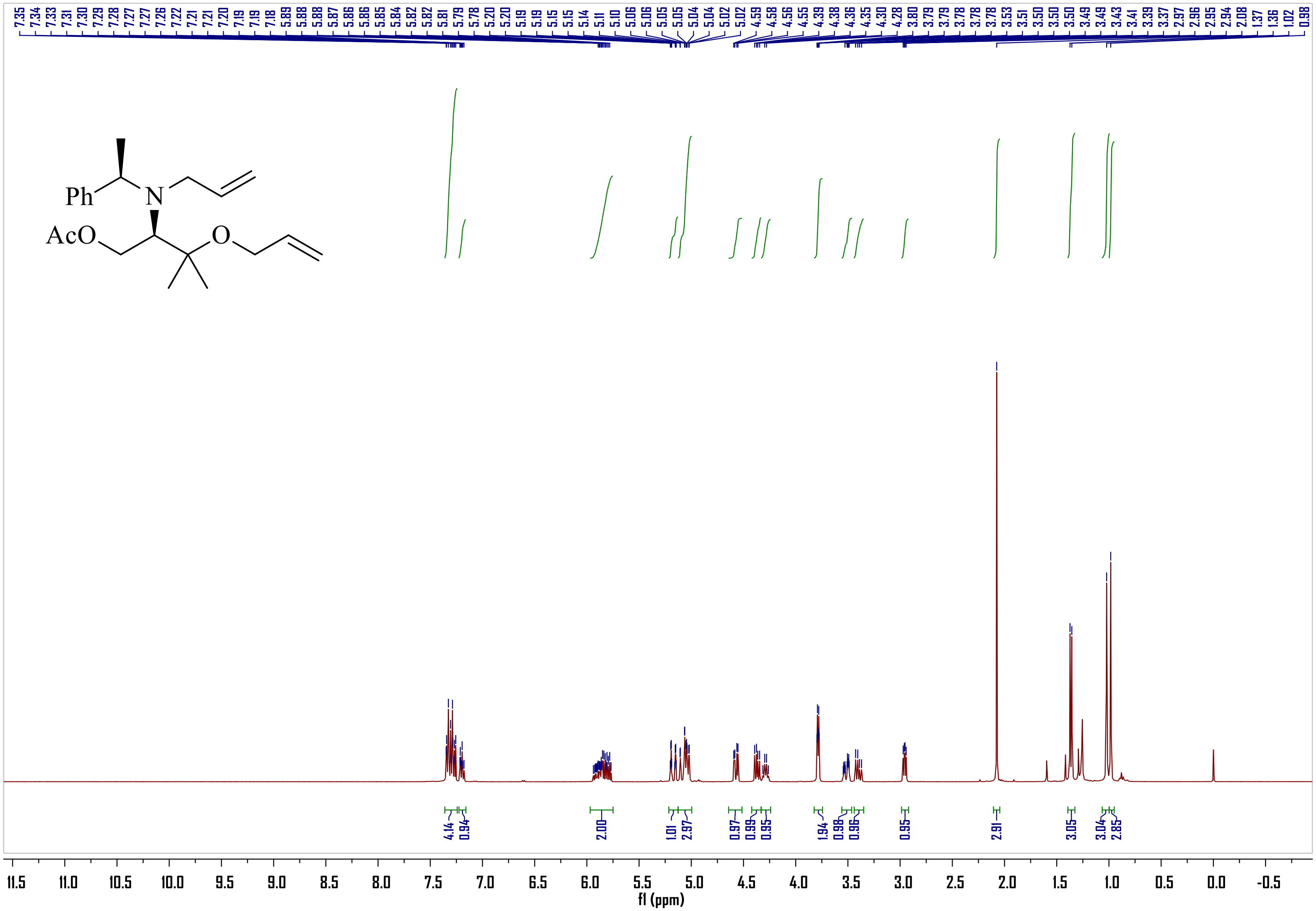

^{13}C NMR spectrum of (R)-2-(allyl((R)-1-phenylethyl)amino)-3-(allyloxy)-3-methylbutyl acetate (12):

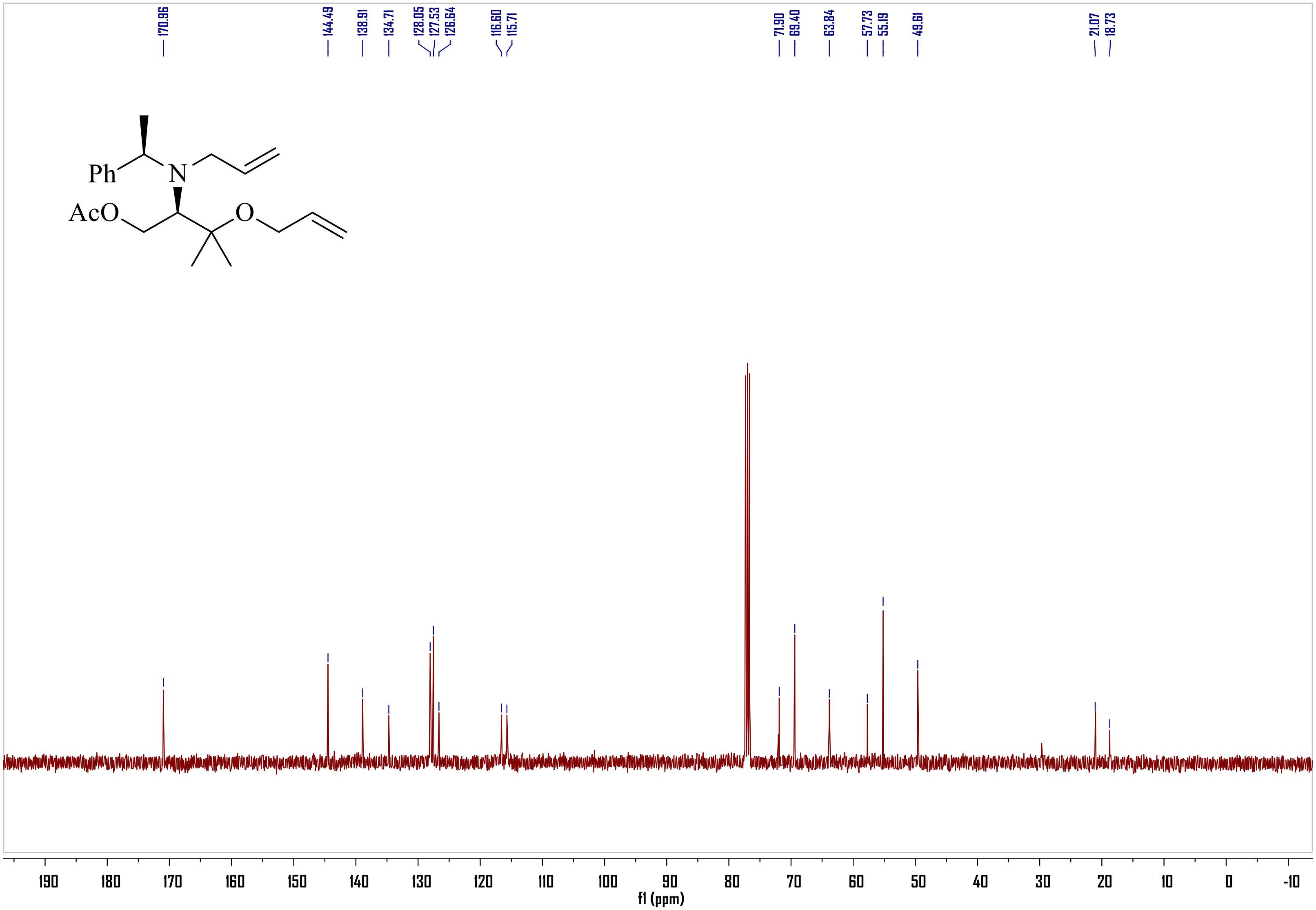

^{1}H NMR spectrum of ((R,Z)-2,2-dimethyl-4-((R)-1-phenylethyl)-3,4,5,8-tetrahydro-2H-1,4-oxazocin-3-yl)methyl acetate (13):

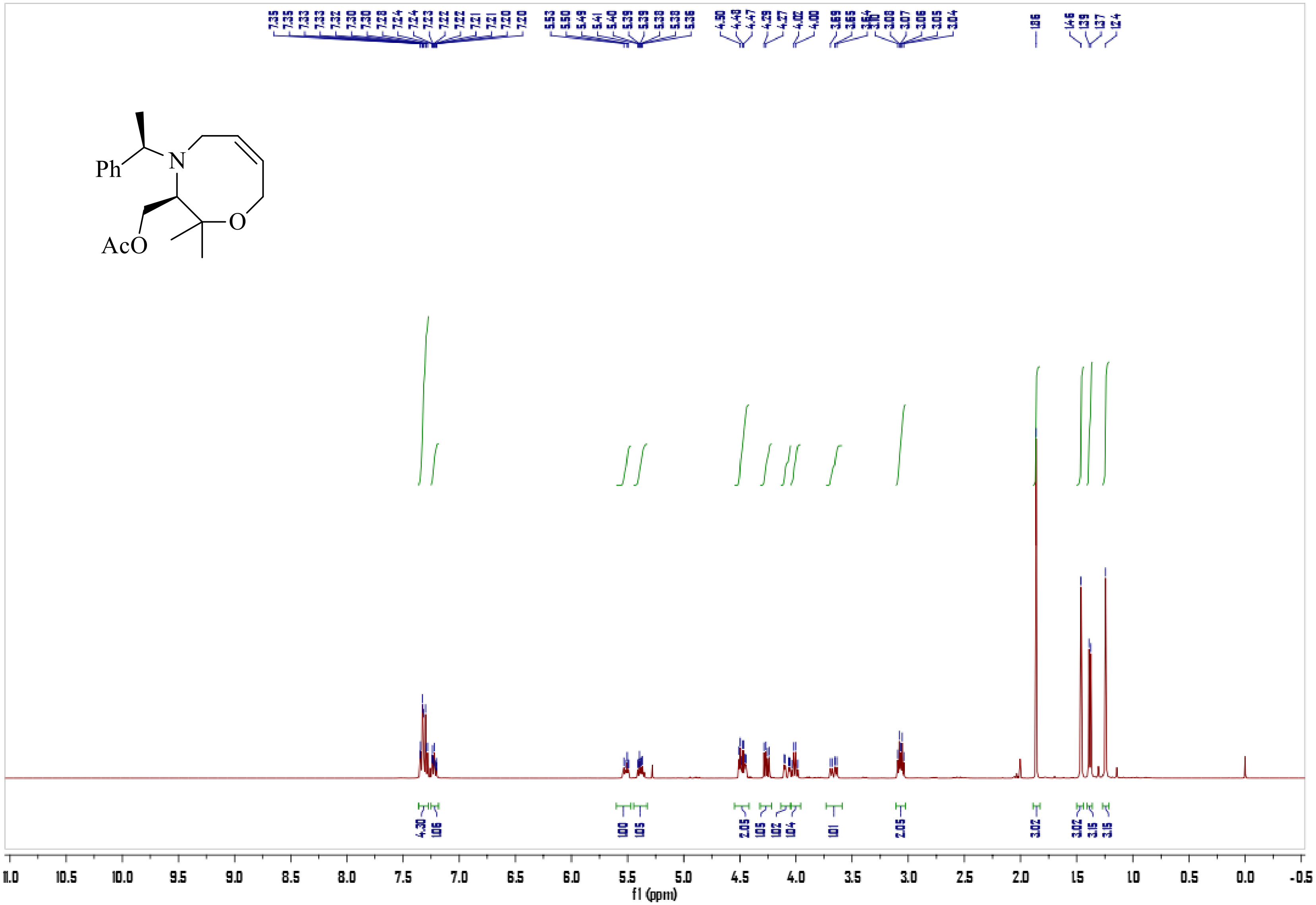

^{13}C NMR spectrum of ((R,Z)-2,2-dimethyl-4-((R)-1-phenylethyl)-3,4,5,8-tetrahydro-2H-1,4-oxazocin-3-yl)methyl acetate (13):

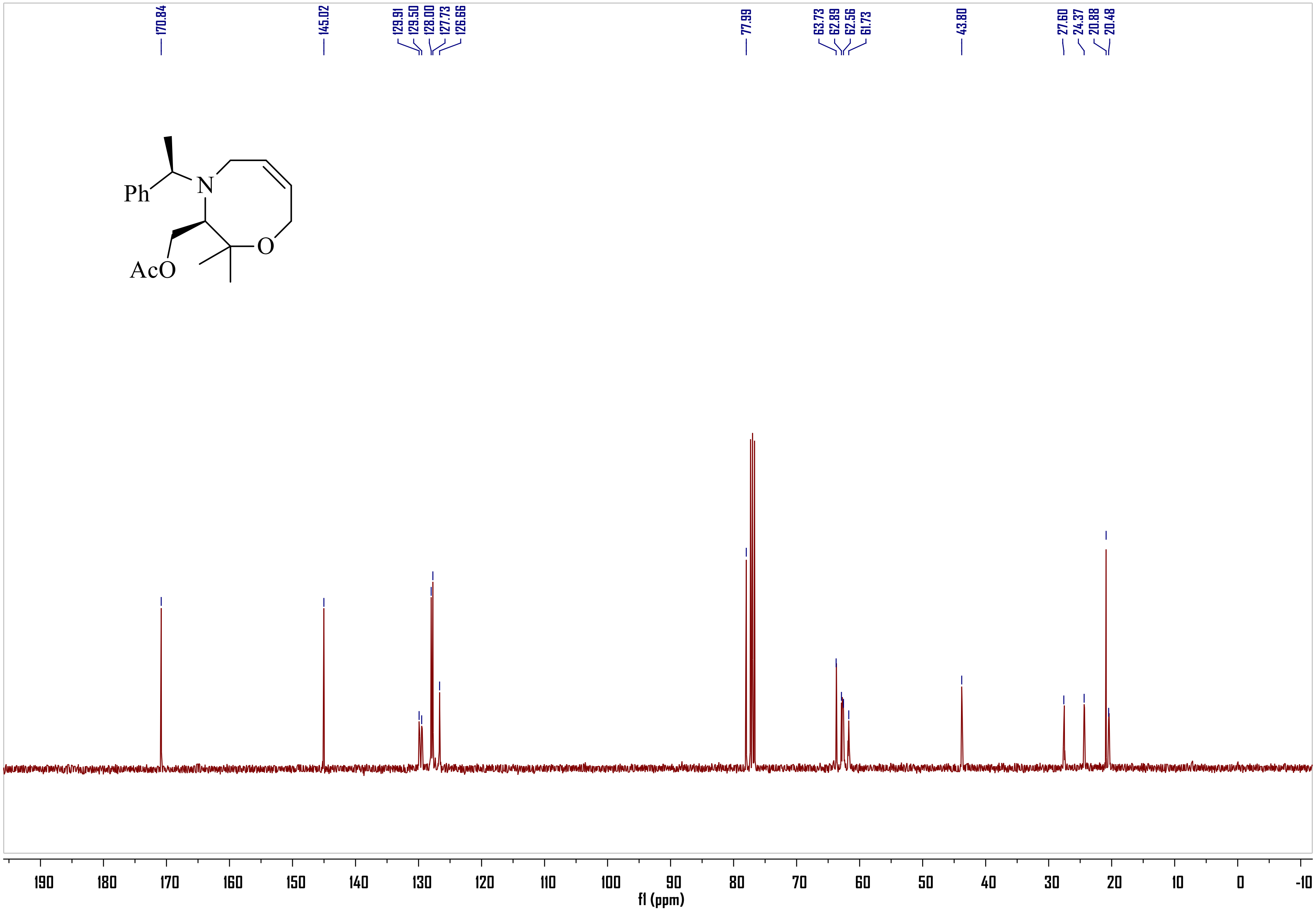

^{1}H NMR spectrum of (R)-tert-butyl 3-(acetoxymethyl)-2,2-dimethyl-1,4-oxazocane-4-carboxylate (14):

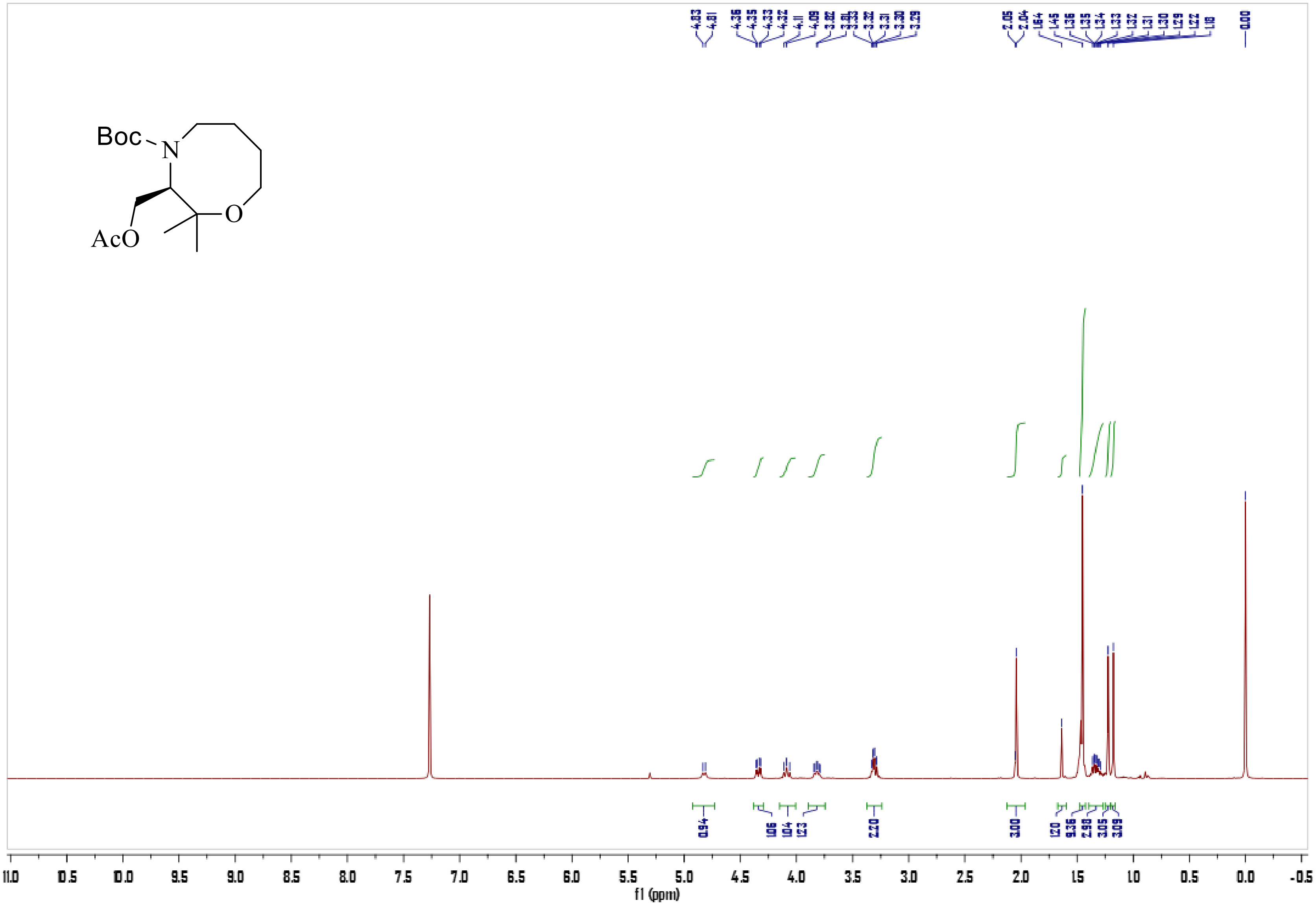

^{13}C NMR spectrum of (R)-tert-butyl 3-(acetoxymethyl)-2,2-dimethyl-1,4-oxazocane-4-carboxylate (14):

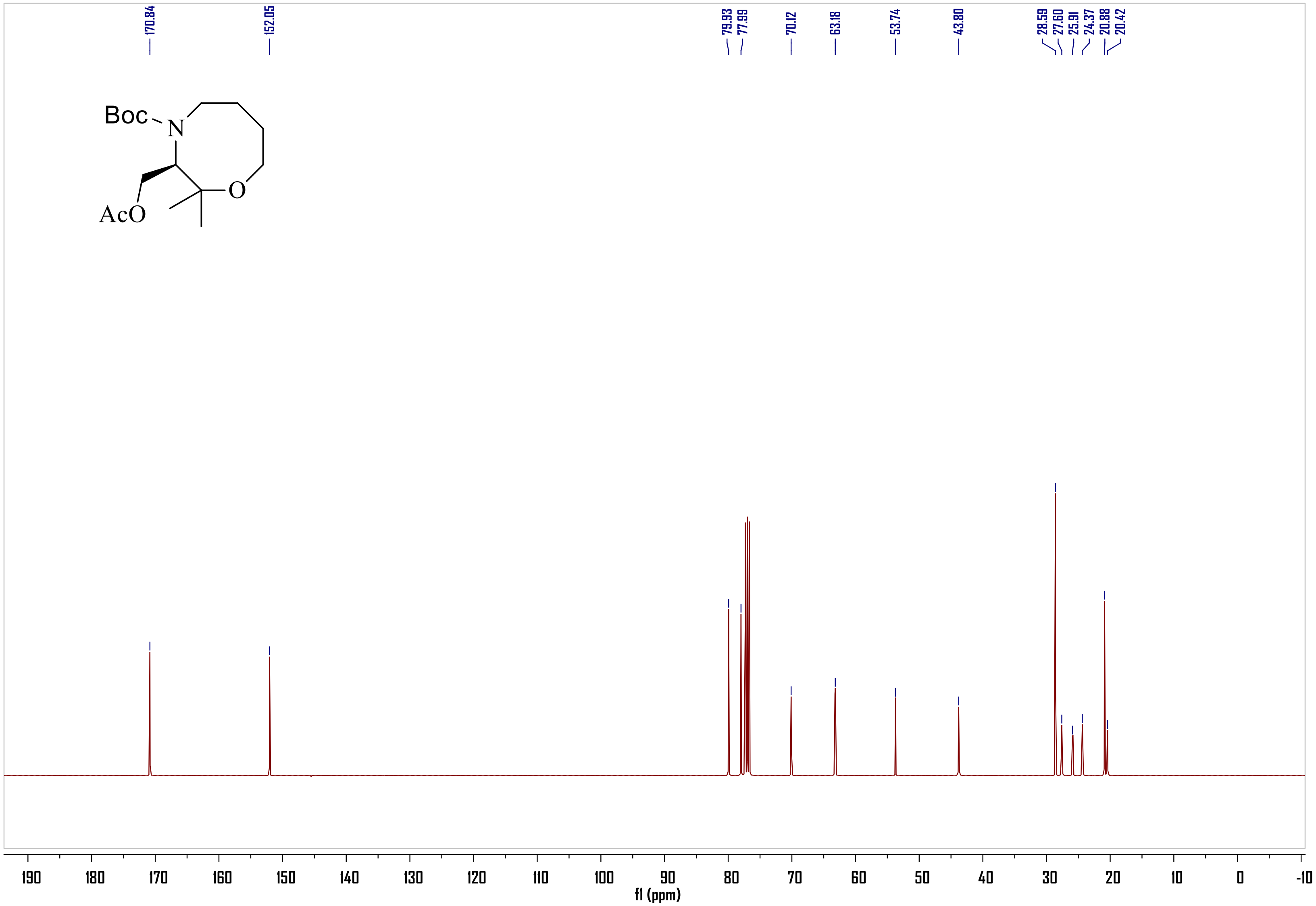

Supplement: Supplementary file 1 [file molecules-26-01703-s001.zip › molecules-1149162-supplementary.pdf]
